# Development of Mass Spectrometry-Based SCFA Analysis Methods in Diverse Samples for Microbiome Research

**DOI:** 10.3390/life16060974

**Published:** 2026-06-09

**Authors:** Chaeeun Park, Md Abdur Rahim, Indrajeet Barman, Hanieh Tajdozian, Youjin Yoon, Sukyung Kim, Mijung Kim, Hoonhee Seo, Ho-Yeon Song

**Affiliations:** 1 Department of Medical Science, Graduate School, Soonchunhyang University, 22, Soonchunhyang-Ro, Sinchang-Myeon, Asan-si 31538, Republic of Korea; 2 K-Microbiome Institute, 813-26 Yisunsin-Daero, Asan-si 31462, Republic of Korea; 3 Department of Microbiology and Immunology, School of Medicine, Soonchunhyang University, 31, Suncheonhyang 6-Gil, Dongnam-Gu, Cheonan-Si 31151, Republic of Korea

**Keywords:** short-chain fatty acids (SCFAs), microbiome, GC-MS/MS, headspace analysis, biological matrices

## Abstract

With the growing interest in the microbiome, short-chain fatty acids (SCFAs) have emerged as key metabolites due to their critical roles in host physiology, including immune regulation, energy homeostasis, and inflammatory control. As a result, the accurate quantification of SCFAs in various biological samples has become increasingly important. However, reliable and standardized methods for measuring SCFAs across different sample types remain underdeveloped, highlighting the need for methodological refinement. To address this need, we optimized two analytical methods, headspace GC-MS and GC-MS/MS, for SCFA quantification. These techniques were applied to a range of biological matrices, including pure microbial cultures, low-abundance animal liver, animal feces, and standardized simulated human fecal samples. The headspace GC-MS approach enables direct analysis with minimal sample preparation, thereby enhancing throughput and ease of use. In contrast, the GC-MS/MS method, involving methanol extraction, alkaline treatment, and derivatization with MTBSTFA, offers superior sensitivity and precision, making it particularly suitable for small-volume and low-abundance samples. Together, these optimized protocols provide robust, sensitive platforms for profiling SCFAs across diverse biological matrices, facilitating a deeper understanding of microbiome–host interactions and supporting future translational applications.

## 1. Introduction

Short-chain fatty acids (SCFAs) are a class of fatty acids characterized by aliphatic tails that generally contain two to six carbon atoms [1]. The short length of their carbon chains gives them a low molecular weight, a principal feature that differentiates them from long-chain fatty acids at both structural and functional levels [2]. Commonly studied SCFAs are acetic acid (C2), propionic acid (C3), butyric acid (C4), and valeric acid (C5) [3].

SCFAs are essential mediators in human physiology [4]. They are predominantly synthesized in the colon via microbial fermentation of dietary fibers and complex carbohydrates [5]. These metabolites function as crucial energy substrates for colonocytes, reduce intestinal pH to suppress pathogenic bacterial proliferation, and influence immune regulation, metabolic homeostasis, lipid metabolism, and satiety signaling [3]. In addition, SCFAs strengthen the integrity of the intestinal barrier and have anti-inflammatory properties mediated by specific physiological pathways [1].

Clinically, SCFAs are recognized for their regulatory effects in a range of diseases, particularly in metabolic syndromes and inflammatory bowel conditions [6]. The concentrations of SCFAs maintain a strong correlation with gut health, and deviations from normal SCFA levels are associated with dysbiosis, obesity, diabetes, Crohn’s disease, and irritable bowel syndrome (IBS) [5]. Moreover, SCFAs can regulate metabolic pathways by reducing adiposity and enhancing insulin sensitivity [7]. Accumulating clinical evidence indicates that SCFA supplementation or increased fiber intake may help prevent or treat these conditions [8].

For the accurate quantification of SCFAs, gas chromatography (GC) and gas chromatography–mass spectrometry (GC-MS) are widely used [9]. GC provides high-resolution separation of volatile compounds, such as SCFAs, depending on their volatility and interaction with the stationary phase. In this context, a study showed the advantages of this approach such as optimized the lyophilization period from 12 h to 3.5 h; disposed of the procedure for precise weight control; lowered the extraction temperature from 25 °C to 4 °C; shortened the extraction time from 45 min to 15 min; and significantly improved the extraction efficiency of acetic acid, propionic acid and butyric acid by 12.91%, 19.95% and 13.08%, respectively [10]. The combination of GC with mass spectrometry (GC-MS) enables precise structural identification and quantitative determination of SCFAs [11], making it a robust approach for detecting SCFAs in biological samples [10].

Nonetheless, both GC and GC-MS methods have inherent limitations [12]. For instance, although SCFAs are volatile, they can be lost during sample pretreatment steps, potentially adversely affecting analytical recovery [12]. Moreover, these techniques are relatively labor-intensive and may not offer sufficient throughput for large-scale studies [13]. The complexity introduced by a variety of compounds present in biological matrices can further hinder both separation and identification during GC-MS analysis [12]. Collectively, these challenges highlight the need for novel analytical strategies that enable more rapid and accurate quantification of SCFAs.

This study addresses these challenges by developing two complementary strategies for robust and efficient SCFA quantification. First, a headspace analysis method was adopted to facilitate rapid, direct assessment without sample pretreatment. Concurrently, a sensitive and selective GC-MS/MS protocol was designed to enable precise qualitative and quantitative analysis of individual SCFAs, incorporating derivatization and tailored extraction steps. Extraction-based GC-MS approaches have been widely applied for SCFA analysis in biological samples. These methods generally involve solvent extraction and chemical derivatization to improve the volatility, stability, and detectability of SCFAs, requiring longer sample preparation times and multiple processing steps [9,14]. In addition to derivatization-based GC-MS/MS approaches, several derivatization-free or minimally derivatized GC-MS workflows have also been developed for SCFA analysis in biological samples [15]. These methods commonly involve sample acidification followed by liquid–liquid extraction using organic solvents, including methyl tert-butyl ether (MTBE) or ethyl acetate, with subsequent direct GC-MS analysis often performed in selected ion monitoring (SIM) mode [16]. Each method offers specific analytical advantages and limitations depending on sample type. For example, early phase studies used acidified water extraction followed by direct GC or GC-MS analysis without extensive chemical derivatization. Regarding this, a study conducted by *Zhao et al. (2005)* applied acidified water extraction with direct GC analysis in colonic and faecal samples from rats and humans [15], showing relatively simple sample preparation and moderate quantification performance for major SCFAs with quantification limit between 2.38 and 30.14 µM. Similarly, another study used ethyl acetate extraction combined with simplified GC-MS analysis for fecal SCFA profiling, indicating an improved simple workflow. In this method, no further cleanup, concentration, and derivatization steps were needed and the extract was directly injected onto the column, showing recoveries ranged between 65 and 105%, with limits of detection between 0.49 and 4.31 μM [16]. Though these independent extraction-based GC-MS/SIM methods provide important practical advantages, reduced sensitivity and higher limits of detection/quantification (LOD/LOQ) was noticed in those methods, particularly compared with derivatization-based GC-MS/MS methods. Additionally, they have limitations in low-abundance samples, volume-sensitive samples or samples containing diverse metabolites and biological contaminants. Recent longitudinal microbiome studies involving preterm infants further highlighted that SCFA concentrations can vary substantially depending on age, disease condition, diet, and sample matrix, emphasizing the need for flexible analytical strategies optimized for different biological conditions [17]. However, the shortcomings of these methods demand other analytical methods for better SCFAs quantification. This study shows that the derivatization-based GC-MS/MS approach enabled selective detection of SCFAs even in highly complex matrices with higher limits of detection. However, both the GC and GC-MS methods improve both data precision and workflow efficiency, allowing users to select the most appropriate technique for their sample type. Ultimately, the analytical framework introduced here is expected to accelerate research and development in microbiome-based probiotics. Furthermore, this platform will not only support microbiome investigations but also enhance mechanistic studies of SCFA physiological functions, providing new perspectives on disease prevention and treatment.

## 2. Materials and Methods

### 2.1. Preparation of Standard Compounds of Short-Chain Fatty Acids

The following standard compounds of short-chain fatty acids were utilized: acetic acid (CAS No. 64-19-7, 99.9%), propionic acid (CAS No. 79-09-4, 99.9%), and butyric acid (CAS No. 107-92-6, 99.7%), procured from TCI (Portland, OR, USA), while valeric acid (CAS No. 109-52-4, 99.4%) was sourced from Sigma-Aldrich (Table 1).

### 2.2. Headspace GC-MS Analysis for Short-Chain Fatty Acid Quantification

#### 2.2.1. Sample Preparation

Samples and standards of short-chain fatty acids were placed in sealed headspace vials to facilitate efficient volatilization and subsequent analysis by GC-MS (Figure S1). Specifically, 2.5 g of sodium chloride (NaCl, CAS No. 7647-14-5; Daejung, Siheung, Republic of Korea), 5 mL of sample, and 1 mL of 2% sulfuric acid (CAS No. 7664-93-9; Daejung, Republic of Korea) were introduced into a 20 mL headspace vial, which was then sealed with a PTFE-lined crimp cap. Standard solutions were produced using this same approach, with acetic acid diluted to 1–1000 μg/mL and the other acids diluted to 1–100 μg/mL in water.

#### 2.2.2. Headspace Instrument Settings

Headspace extraction was conducted using a TurboMatrix Headspace sampler with stringent control over temperature, pressurization, and injection parameters (Figure S2). The needle, headspace sampler transfer line, and oven temperatures were established at 150 °C, 160 °C, and 90 °C, respectively. Each vial underwent equilibration at 90 °C for 20 min (thermostat time). The pressurization period was set to 3.0 min, the withdrawal duration to 0.3 min, and a sample injection volume of 0.16 mL was used. Injection time was automatically determined based on the carrier gas flow rate, and the GC cycle duration was set to 20 min.

#### 2.2.3. GC Conditions

Gas chromatographic analysis utilized an Elite-FFAP column and a temperature-controlled oven to achieve efficient separation of SCFAs (Figure S3). Samples were injected into a Perkin Elmer Clarus 690 GC in split mode (split ratio 50:1) at an injection temperature of 250 °C. The oven was initially held at 120 °C, increased at 5 °C/min, and held at 200 °C. Helium served as the carrier gas with a flow rate of 1.00 mL/min.

#### 2.2.4. Mass Spectrometry Conditions

Detection was performed by electron ionization mass spectrometry in SIM mode, using predefined ion values specific to each SCFA (Figure S4). To minimize interference from early-eluting solvents, a solvent delay of 2.3 min was implemented. The ion source temperature was set to 250 °C. Quantification was based on SIM mode monitoring *m*/*z* 43 and 45 for acetic acid, 45 and 74 for propionic acid, and 60 and 73 for both butyric and valeric acids.

#### 2.2.5. Sample Injection Sequence

Sample injection was programmed in a sequence of blanks, calibration standards, and samples to maintain analytical stability and minimize carryover (Figure S5). The sequence began with a base vial to stabilize the system, followed by a series of standard mixtures at increasing concentrations. A second blank was injected to eliminate any remaining analytes, after which biological samples were analyzed consecutively. All procedures were fully automated using the TurboMatrix autosampler.

#### 2.2.6. Summary of Instrumental Conditions

All GC-MS parameters, including instrument configuration, column characteristics, temperature settings, flow rates, and ion information, are summarized in a reference table (Table S1). This comprehensive table provides a concise overview of all operating parameters used for headspace GC-MS analysis of SCFAs.

### 2.3. GC-MS/MS Analysis

#### 2.3.1. Materials

For sample pretreatment and derivatization, the following reagents were employed: HPLC-grade methanol (CAS No. 67-56-1, J.T. Baker, Phillipsburg, NJ, USA), methoxyamine hydrochloride (CAS No. 593-56-6, Sigma-Aldrich, St. Louis, MO, USA), pyridine (CAS No. 110-86-1, Samchun, Daejeon, Republic of Korea), and MTBSTFA (N-tert-Butyldimethylsilyl-N-methyltrifluoroacetamide, CAS No. 77377-52-7, Sigma-Aldrich, USA). For sample processing and derivatization, the instruments used included the MG-2200 nitrogen concentrator (EYELA, Tokyo, Japan), the MICRO 17TR centrifuge (Hanil, Gimpo, Republic of Korea), the ME204 analytical balance (Mettler Toledo, Greifensee, Zürich, Switzerland), and the HyperVAC VC2200 vacuum concentrator (Hanil, Korea). Quantitative determinations were conducted using a GC-MS/MS system consisting of the GC-2010 Plus and TQ8040 triple quadrupole mass spectrometer (Shimadzu, Kyoto, Japan), fitted with a DB-5MS capillary column (30 m × 0.25 mm, 0.25 μm film thickness; Agilent, Santa Clara, CA, USA).

#### 2.3.2. Sample Preparation and Derivatization

The step-by-step protocol for SCFA pretreatment is depicted in Figure S6. In brief, 20 mg of sample was combined with 30 μL of 0.1 M NaOH and 430 μL methanol in an Eppendorf tube, followed by incubation at –20 °C for 20 min. The mixture was then centrifuged at 22,000× *g* for 10 min at 4 °C, and 450 μL of the supernatant was transferred and dried under vacuum at 37 °C. To improve derivatization consistency prior to silylation, 40 μL of 20 mg/mL methoxyamine hydrochloride in pyridine was added to the dried residue, and the mixture was incubated at 60 °C for 90 min [18]. Thereafter, 60 μL of MTBSTFA was added, and the solution was incubated at 60 °C for 30 min. Following a subsequent centrifugation (22,000× *g*, 10 min, 4 °C), 70 μL of the supernatant was mixed with 140 μL of pyridine and used as the final solution for GC-MS/MS analysis. For quantification, standard stock solutions (1000 μg/mL) of acetic acid, propionic acid, butyric acid, and valeric acid were prepared by dissolving 100 mg of each analyte in methanol, then adjusting the volume to 100 mL. Serial dilutions were performed to produce working standards at 1, 2, 5, 10, 20, 50, and 100 ng/mL concentrations. All standards were subjected to the identical derivatization procedure as the experimental samples [19].

#### 2.3.3. Instrumentation and GC-MS/MS Conditions

The analytical platform for SCFA determination consisted of a GC-2010 Plus coupled with a split/splitless injector, a temperature-controlled oven, and a TQ8040 triple quadrupole mass spectrometer (Shimadzu, Japan). Details of the instrumental setup are outlined in Figure S7. An autosampler was employed for all sample and standard injections, performed at 250 °C with a 5:1 split ratio. The GC oven was programmed to start at 60 °C (1 min hold), then ramp at 10 °C/min to 200 °C, then at 30 °C/min to 325 °C, with a final hold for 5 min. Helium was used as the carrier gas at a flow rate of 1 mL/min. After separation, analytes were transferred through a transfer line maintained at 290 °C and ionized in the source at 230 °C. The mass spectrometer operated in MRM (Multiple Reaction Monitoring) mode with specific transitions set for each analyte.

#### 2.3.4. Sequence Analysis and Method Setup

The sample sequence was designed to minimize carryover and maintain stable instrument performance (Figure S8). The injection protocol started with a blank vial to equilibrate the GC-MS/MS system, followed by runs of standard mixtures with increasing concentration from lowest to highest. To mitigate contamination by residual analytes from standard vials, an additional blank was injected after the standards. Experimental samples were then injected sequentially, with base matrix injections interspersed to assess possible carryover. All procedures were executed using the GC-2010 Plus autosampler to ensure consistency and full automation. Instrument conditions were set according to precursor–product ion transitions and specific collision energy parameters for each SCFA.

#### 2.3.5. Summary of Operating Parameters

The general operating parameters for the GC-MS/MS are outlined in Table S2. SCFA quantification was conducted using optimized MRM transitions: acetic acid was monitored at 117 → 75, 118 → 76, and 117 → 71; propionic acid at 131 → 75, 131 → 112, and 131 → 83; butyric acid at 145 → 140, 145 → 75, and 145 → 93; and valeric acid at 147 → 73, 189 → 147, and 148 → 60. The use of optimized transitions and established GC parameters enabled a reliable, reproducible determination of SCFAs in complex biological matrices.

### 2.4. Strategy Assessment for Quantification and Description of Microbiome Samples

#### 2.4.1. Assessment of a Strategy for Quantification Method Selection

In this study, we evaluated two analytical methods for quantifying short-chain fatty acids (SCFAs) to identify the most suitable method for each sample type. Due to the Headspace method’s inherent need for larger sample volumes, we employed culture media samples for comparative experiments, as adequate quantities could be obtained. Initially, the sensitivity of each instrument was assessed by determining its limit of detection. To serve this purpose, culture media samples were analyzed to quantify SCFA using a headspace GC-MS method, employing a TurboMatrix Headspace sampler and Perkin Elmer Clarus 690 GC-MS system with an Elite-FFAP column under split mode (50:1). Samples were prepared with sodium chloride and sulfuric acid, equilibrated at 90 °C for 20 min, and analyzed in SIM mode targeting specific *m*/*z* values for each SCFA. At the same time, culture media samples were analyzed using a GC-MS/MS system after derivatization with methoxyamine and MTBSTFA. Samples were analyzed in MRM mode using specific ion transitions, employing a DB-5MS column under a temperature program from 60 °C to 325 °C. In both cases, calibration standards and blanks were included in the injection sequence to ensure analytical accuracy and minimize carryover. A pure microbial culture of *Lacticaseibacillus rhamnosus* and standardized simulated human fecal samples were used in this study, and detailed descriptions of each sample type are provided in the following sections.

#### 2.4.2. Description of Samples Used in Microbiome Analysis

To evaluate the applicability and robustness of quantification methods across diverse microbiome-related contexts, this study used four distinct types of biological samples: pure microbial cultures, low-abundance animal liver, animal feces, and standardized simulated human fecal samples. These samples were selected to represent a broad spectrum of matrix complexity, microbial load, and physiological relevance, thereby allowing a comprehensive assessment of method performance under varied analytical conditions. Each sample type reflects representative use cases in microbiome research, ranging from controlled microbial systems to complex biological environments, with detailed methodologies provided in the referenced studies and key aspects summarized below [20,21,22]. *L. rhamnosus* represented pure microbial cultures. A 30 µL aliquot of the bacterial stock was inoculated into 30 mL of MRS medium (BD Difco, Franklin Lakes, NJ, USA) at a final concentration of 0.1% (*v*/*v*) and incubated at 37 °C for 18 h. Bacterial growth was monitored by measuring optical density at 600 nm (OD600) using a spectrophotometer (DR 1900, Hach, Loveland, CO, USA). When the culture reached an OD of 1.0, it was centrifuged at 4000 rpm for 10 min, and the supernatant was collected for short-chain fatty acid (SCFA) analysis by headspace or GC-MS/MS. Alternatively, samples were stored at −80 °C until further analysis. To select appropriate analytical methods based on sample type, pure microbial cultures of *L. rhamnosus* and two strains of *Pediococcus acidilactici* were analyzed using the headspace method. All strains were cultured under the same conditions described above. For GC-MS/MS analysis, two strains of *Ruminococcus bromii* and *Rutibacterium gallinarum* were used. Each culture (30 µL) was inoculated into 30 mL of modified YCFA medium (Anaerobe Systems, Morgan Hill, CA, USA) and incubated at 37 °C for 48–72 h until an OD of 1.0 was reached. After incubation, the culture supernatants were collected and analyzed as described above. Low-abundance animal liver samples were obtained from C57BL/6J mice. Mice were divided into control, streptozotocin (STZ)-induced diabetic, butyrate-treated, and probiotic-treated groups, and liver tissues were collected for analysis. Animal fecal samples were obtained from BALB/c mice, and fecal samples were collected over time following *Klebsiella pneumoniae* infection. Standardized simulated human fecal samples were generated using a human gut microbiota simulation system (ProDigest BVBA (SHIME^®^), Ghent, East Flanders, Belgium), which simulates the physicochemical and microbial conditions of the human colon in vitro. Samples were collected from the descending colon over time.

## 3. Results

### 3.1. Results of Method Validation for Quantitative Analysis of SCFAs Using Headspace GC-MS

The method validation of headspace GC-MS for quantifying short-chain fatty acids (SCFAs) is detailed in a table summarizing calibration linearity, detection sensitivity, spike-in recovery, retention times, and selected ion specificity (Table 2). Recovery (%) was calculated as: [(C_spiked − C_unspiked)/C_added] × 100. The elevated spike-in recovery values may be associated with matrix-enhanced responses arising from endogenous metabolite abundance and derivatization-dependent analytical variability in complex biological samples. Calibration curves for acetic acid, propionic acid, butyric acid, and valeric acid exhibited robust linearity, with R^2^ values in the range of 0.995150 to 0.997167 (Figure S9). These findings demonstrate that the instrument response remained strongly proportional to SCFA concentration across the examined range. Detection sensitivity was evaluated using chromatograms obtained at two concentrations (200 μg/mL and 1 μg/mL). At 200 μg/mL, distinct peaks for all four SCFAs were observed, each displaying high signal-to-noise (S/N) ratios. At 1 μg/mL, butyric acid and valeric acid maintained quantifiable peaks. In contrast, acetic acid and propionic acid exhibited noticeably weaker signals, which suggests decreased sensitivity for these analytes at lower concentrations (Figure S10). Based on the chromatographic profiles, 1 μg/mL was determined as the minimum reliable concentration detectable by headspace GC-MS. Retention times were observed as 3.96 min for acetic acid, 4.80 min for propionic acid, 5.87 min for butyric acid, and 7.41 min for valeric acid, demonstrating consistent compound separation and high reproducibility. Spike-in recovery analysis involved adding standard SCFA solutions to biological matrices (Matrix A and B) at a 1:1 volume ratio. Recovery (%) was calculated as [(C_spiked − C_unspiked)/C_added] × 100, where C_spiked represents the measured concentration in the spiked sample, C_unspiked represents the endogenous concentration in the unspiked sample, and C_added represents the nominal spiked concentration. The measured concentrations of acetic acid in spiked samples were 162.91 μg/mL (Matrix A) and 156.10 μg/mL (Matrix B), corresponding to recovery rates of 102.96% and 103.53%, respectively. For propionic acid, butyric acid, and valeric acid, a spike-in with a 500 μg/mL standard was performed, and recovery rates in both matrices ranged from 95.46% to 138.83%, demonstrating the method’s reliability under dilution. Characteristic fragment ions for each SCFA were determined using selected ion monitoring (SIM). Acetic acid was detected at *m*/*z* 43 and 45; propionic acid at *m*/*z* 45 and 74; butyric acid at *m*/*z* 60 and 73; and valeric acid at *m*/*z* 60 and 73 (Figure S11), confirming the method’s selectivity and supporting the specific identification of each SCFA. The observed recovery rates indicate that the headspace GC-MS method provides relatively reliable and reproducible SCFA quantification in biological matrices. In addition, the detection limit of approximately 1 μg/mL suggests that the method is suitable for metabolomics studies. In summary, the headspace GC-MS method demonstrated excellent calibration linearity, adequate sensitivity at low concentrations, robust recovery in spike-in experiments, and high specificity in ion detection, supporting its application for SCFA quantification in biological matrices.

### 3.2. Optimization of MRM-Based Quantitative Analysis of SCFAs Using GC-MS/MS

GC-MS/MS analysis demonstrated greater sensitivity than headspace GC-MS, enabling quantification down to 1 ng/mL (Table 3). All four short-chain fatty acids (SCFAs)—acetic acid, propionic acid, butyric acid, and valeric acid—displayed excellent linearity, with calibration coefficients (R^2^) greater than 0.995 (Figure S12). Chromatographic analysis confirmed the retention times, identifying acetic acid at 4.398 min, propionic acid at 5.598 min, butyric acid at 6.948 min, and valeric acid at 7.248 min. The elution sequence matched that of the headspace GC-MS analysis (Figure S13). Retention times were validated using the instrument’s table view function, which specified the actual measurement intervals and anticipated peak times for each compound (Figure S14). Candidate precursor ions for multiple reaction monitoring (MRM) were determined using Q3 full-scan mode. Spectral data confirmed that acetic acid produced dominant ions at *m*/*z* 75 and 117, propionic acid at *m*/*z* 75 and 131, butyric acid at *m*/*z* 75 and 145, and valeric acid at *m*/*z* 73, 147, and 189 (Figure S15). These ions served as the basis for selecting transitions for MRM optimization. Collision energy (CE) optimization was performed using a batch-mode product-ion scan, with CE adjusted from 3 to 45 V to assess the yield for each precursor–product ion combination (Figure S16). This approach identified the CE that produced the highest product-ion intensity for each transition. The associated intensity profiles at varying CE settings are presented graphically for each SCFA (Figure S17). These plots illustrated unique CE optima for each precursor–product ion pair. For instance, the transition 117 → 75 (acetic acid) exhibited optimal response at 9 V, while 147 → 73 (valeric acid) was optimal at 15 V. Comprehensive CE values and peak intensity data for each MRM transition are reported numerically (Figure S18). Acetic acid transitions showed optimal performance at 117 → 75 (CE 9 V), 118 → 76 (CE 6 V), and 117 → 71 (CE 12 V). Propionic acid achieved maximum sensitivity at 131 → 75 (CE 21 V), 131 → 112 (CE 6 V), and 131 → 83 (CE 6 V). For butyric acid, transitions were optimized at 145 → 140 (CE 27 V), 145 → 75 (CE 3 V), and 145 → 93 (CE 6 V). Valeric acid transitions displayed the highest responses at 147 → 73 (CE 15 V), 189 → 147 (CE 3 V), and 148 → 60 (CE 36 V). Collectively, these data establish a reliable set of verified transitions and CE parameters for precise SCFA quantification by GC-MS/MS.

### 3.3. Application of Quantification Strategy According to Sample Type

**Figure 1:**
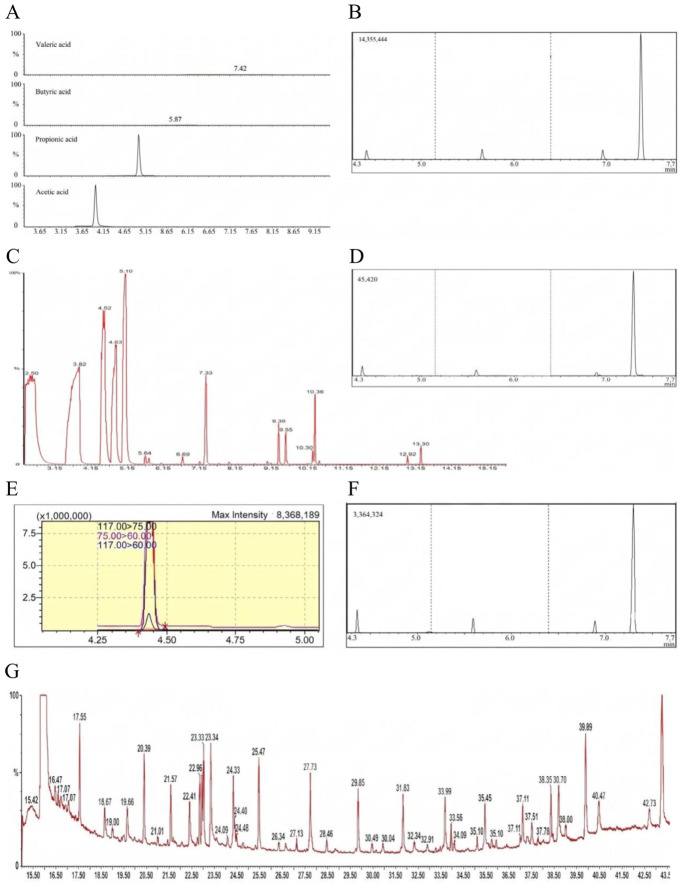
**Comparison of Headspace and GC-MS/MS Methods for Selecting Appropriate Analytical Strategies for Various Biological Samples.** (**A**) The culture medium of *Lacticaseibacillus rhamnosus* diluted to 1 ng/mL was analyzed using the headspace method; however, insufficient sensitivity prevented the identification of the compound peaks. This diluted sample was prepared from a stock solution whose concentration had been determined by headspace analysis. (**B**) The same diluted culture medium was analyzed by GC-MS/MS, enabling reliable identification of compound peaks. All relevant peaks were clearly detected, indicating successful identification even without full quantification. (**C**) Headspace analysis of the undiluted culture medium was complicated by high levels of impurities, which hindered detection of the target compounds. (**D**) In contrast, GC-MS/MS analysis of the same undiluted sample minimized interference from surrounding peaks, enabling accurate identification of target compounds. (**E**) When the sample concentration exceeds 200 µg/mL during GC-MS/MS analysis, signal saturation occurs, reducing quantification reliability. Optimal results are generally obtained at concentrations below 100 µg/mL. (**F**) Analysis of standardized simulated human fecal samples using GC-MS/MS enabled selective detection of the target compound peaks, even in complex sample matrices. (**G**) On the other hand, the headspace method resulted in reduced accuracy due to high levels of interfering impurities, which impeded peak identification.

To determine the appropriate analytical method for SCFA quantification across different biological sample types, the performance of the headspace and GC-MS/MS methods was compared based on analyte concentration and sample purity (Figure 1). Due to the large sample volumes required by the headspace method, culture medium samples were selected as a model matrix for comparative analysis. When the *L. rhamnosus* culture medium was diluted to 1 ng/mL, the headspace method failed to detect peaks due to insufficient sensitivity (Figure 1A). In contrast, GC-MS/MS analysis of the same sample enabled reliable identification of all major SCFA peaks, demonstrating high sensitivity and clear detection without the need for full quantification (Figure 1B).

The impact of sample purity was further assessed using undiluted culture medium. Headspace analysis revealed numerous interfering peaks that hindered the identification of target compounds (Figure 1C). In contrast, GC-MS/MS minimized interference and enabled accurate detection of SCFAs in the same sample (Figure 1D). Quantification performance of GC-MS/MS was also influenced by analyte concentration. Signal saturation was observed at sample concentrations above 200 µg/mL, resulting in reduced quantification accuracy. Optimal results were obtained at concentrations below 100 µg/mL (Figure 1E). To evaluate analytical performance in complex matrices, standardized simulated human fecal samples were analyzed.

GC-MS/MS selectively detected SCFAs with high precision, even in the presence of impurities (Figure 1F). Conversely, headspace analysis produced extensive interference, impeding the identification of target peaks (Figure 1G). These findings indicate that the headspace method, while advantageous for its simplicity and speed, is limited by low sensitivity and vulnerability to matrix interference. It is thus more suitable for high-concentration, low-impurity samples such as culture media. By contrast, GC-MS/MS enables highly sensitive and selective detection of SCFAs, even in small-volume or highly impure samples, making it a more robust approach for comprehensive SCFA profiling across diverse biological matrices.

### 3.4. Evaluation of SCFA Quantification in Culture Media Samples Using the Headspace Method

Quantitative analysis by headspace GC-MS revealed that SCFAs were detected in all bacterial cultures analyzed (Figure S19). The standard solution contained four SCFAs—acetic acid, propionic acid, butyric acid, and valeric acid—each at a concentration of 500 µg/mL. All four SCFAs were clearly separated and detected in the standard chromatogram (Figure S19A). In *P. acidilactici* cultures, acetic acid, butyric acid, and valeric acid were quantified at concentrations of 2376.3, 282.1, and 262.2 µg/mL, respectively, based on input concentrations of 102.6179, 12.2066, and 11.3809 µg/mL (Figure S19B). In another *P. acidilactici* strain, the same three SCFAs were detected at concentrations of 2694.4, 287.5, and 259.3 µg/mL, respectively, corresponding to input concentrations of 108.0477, 11.5522, and 10.4520 µg/mL (Figure S19C). In *L. rhamnosus* cultures, the same SCFAs were quantified at concentrations of 5185.1, 276.7, and 244.7 µg/mL, respectively, based on input concentrations of 218.0958, 11.6636, and 10.3428 µg/mL (Figure S19D). Propionic acid was not detected or was below the limit of quantification in all cases. These results demonstrate that the headspace GC-MS method established in this study enables reproducible and reliable quantification of SCFAs across different microbial culture samples.

### 3.5. SCFA Quantification in Microbiome-Associated Samples Using GC-MS/MS

**Figure 2:**
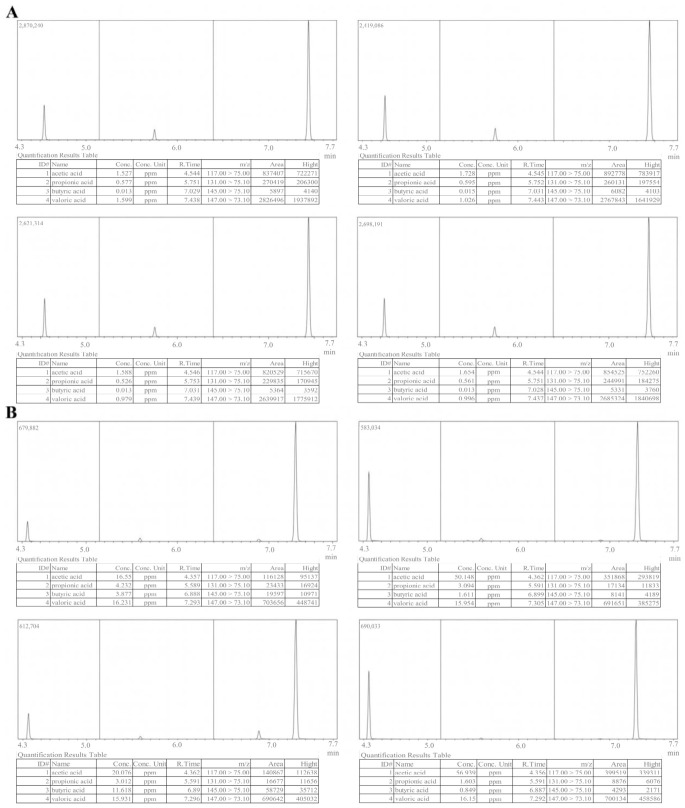

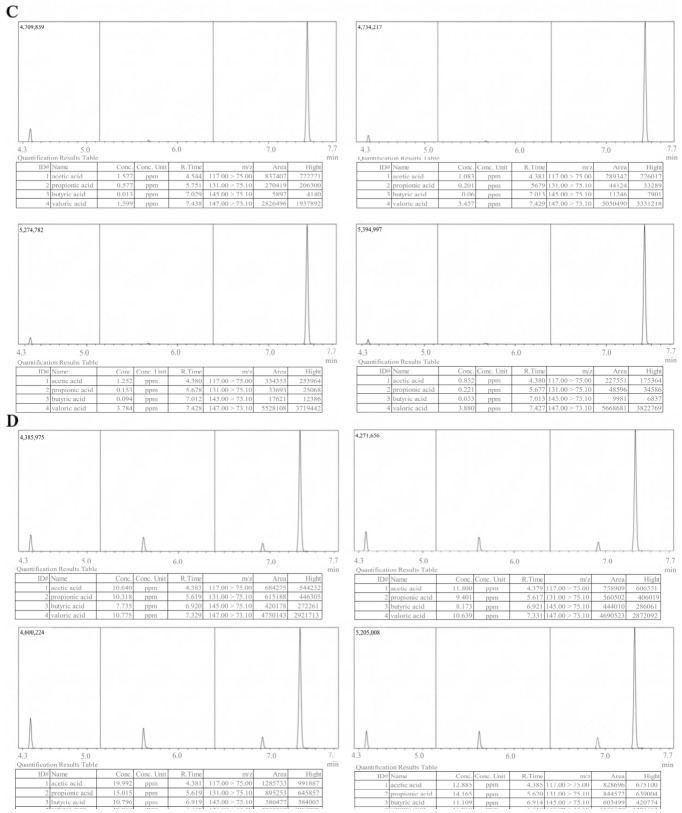
GC-MS/MS-based quantification of short-chain fatty acids (SCFAs) across diverse microbiome sample types. Acetic acid, propionic acid, butyric acid, and valeric acid were quantified in pure microbial cultures, low-abundance animal liver samples, animal feces, and standardized simulated human fecal samples. (**A**) In pure microbial cultures, results are presented for the control medium, two strains of *Ruminococcus bromii*, and *Rutibacterium gallinarum*, in that order. (**B**) In low-abundance animal liver samples, results are shown for control mice, STZ-induced diabetic mice, and diabetic mice treated with butyrate or probiotics, respectively. (**C**) In animal feces, results represent samples collected from mice at days 2 to 8 following Klebsiella pneumoniae infection, in chronological order. (**D**) In standardized simulated human fecal samples, results are shown for descending colon samples collected from the stabilization phase after fecal inoculation through week 6.

Quantitative analysis using the GC-MS/MS method revealed the amount of short-chain fatty acids (SCFAs) detected in diverse biological samples (Figure 2, Table S3).

Pure bacterial cultures were analyzed to determine the amounts of acetic acid, propionic acid, butyric acid, and valeric acid (Figure 2A). In sample 1 (control group), the concentrations of these acids were 152.7 µg/mL, 55.7 µg/mL, 1.3 µg/mL, and 159.9 µg/mL, based on an input of 1.527, 0.557, 0.013, and 1.599, respectively. In sample 2 (culture of *R. bromii* strain 1), concentrations of these acids were 172.8 µg/mL, 59.5 µg/mL, 1.5 µg/mL, and 102.6 µg/mL, based on an input of 1.728 µg/mL, 0.595 µg/mL, 0.015 µg/mL, and 1.026 µg/mL, respectively. In sample 3 (culture of *R. bromii* strain 2), concentrations of these acids were 165.4 µg/mL, 56.1 µg/mL, 1.3 µg/mL, and 99.6 µg/mL, based on an input of 1.654 µg/mL, 0.561 µg/mL, 0.013 µg/mL, and 0.996 µg/mL, respectively. Sample 4 (culture of *R. gallinarum*) yielded the concentrations of acetic acid, propionic acid, butyric acid, and valeric acid, which were 158.8 µg/mL, 52.6 µg/mL, 1.3 µg/mL, and 97.9 µg/mL, respectively, based on an input of 1.588 µg/mL, 0.526 µg/mL, 0.013 µg/mL, and 0.979 µg/mL, respectively. Overall, these findings demonstrate robust and reproducible SCFA quantification in pure microbial cultures.

SCFAs were also quantified in low-abundance animal liver samples (Figure 2B). For this purpose, animals were treated under different conditions, and livers were harvested to measure SCFAs. In sample 1 (control group), the measurements of acetic acid, propionic acid, butyric acid, and valeric acid were 1655.6 µg/mL, 423.2 µg/mL, 387.7 µg/mL, and 1623.1 µg/mL based on an input of 16.56 µg/mL, 4.232 µg/mL, 3.877 µg/mL, and 16.231 µg/mL, respectively. In sample 2 (STZ-treated group), the concentrations of these acids were 5014.80 µg/mL, 309.40 µg/mL, 161.10 µg/mL, and 1595.4 µg/mL based on an input of 50.148 µg/mL, 3.094 µg/mL, 1.611 µg/mL, and 15.954 µg/mL, respectively. In sample 3 (butyrate-treated group), the measurement of these acids was 2007.60 µg/mL, 301.20 µg/mL, 1161.80 µg/mL, and 1593.90 µg/mL based on an input of 20.076 µg/mL, 3.012 µg/mL, 11.618 µg/mL, and 15.931 µg/mL, respectively. In sample 4 (probiotics-treated group), the concentration of acetic acid, propionic acid, butyric acid, and valeric acid was 5693.9 µg/mL, 160.30 µg/mL, 84.90 µg/mL, and 1615.00 µg/mL, respectively, based on an input of 56.939 µg/mL, 1.603 µg/mL, 0.849 µg/mL, and 16.150 µg/mL, respectively. These findings demonstrate reliable SCFA quantification in low-abundance samples.

Moreover, various animal fecal samples were utilized to measure all SCFAs using the GC-MS/MS approach (Figure 2C). Sample 1 (infection day 2) shows the presence of acetic acid, propionic acid, butyric acid, and valeric acid at 220.00 µg/mL, 26.80 µg/mL, 7.80 µg/mL, and 350.60 µg/mL, respectively, based on an input of 2.200 µg/mL, 0.268 µg/mL, 0.078 µg/mL, and 3.506 µg/mL, respectively. In Sample 2 (infection day 6), the concentrations of these acids were 108.30 µg/mL, 20.10 µg/mL, 6.00 µg/mL, and 345.70 µg/mL based on an input of 1.083 µg/mL, 0.201 µg/mL, 0.060 µg/mL, and 3.457 µg/mL, respectively. In Sample 3 (infection day 7), the measurements of these acids were 125.2 µg/mL, 15.30 µg/mL, 9.40 µg/mL, and 378.4 µg/mL based on an input of 1.252 µg/mL, 0.153 µg/mL, 0.094 µg/mL, and 3.784 µg/mL, respectively. Sample 4 (infection day 8) yielded the concentrations of acetic acid, propionic acid, butyric acid, and valeric acid, which were 85.20 µg/mL, 22.10 µg/mL, 5.30 µg/mL, and 388.00 µg/mL, respectively, based on an input of 0.852 µg/mL, 0.221 µg/mL, 0.053 µg/mL, and 3.880 µg/mL, respectively. Overall, these results demonstrate reliable and sensitive SCFA quantification in complex animal fecal matrices, enabling detection of subtle differences across conditions.

Furthermore, standardized simulated human fecal samples were analyzed (Figure 2D). Sample 1 (initial stage) shows the presence of acetic acid, propionic acid, butyric acid, and valeric acid at 1064.00 µg/mL, 1031.80 µg/mL, 773.50 µg/mL, and 1077.50 µg/mL, respectively, based on an input of 10.640 µg/mL, 10.318 µg/mL, 7.735 µg/mL, and 10.775 µg/mL, respectively. In Sample 2 (early stage), the concentrations of these acids were 1180.00 µg/mL, 940.10 µg/mL, 817.30 µg/mL, and 1063.90 µg/mL based on an input of 11.800 µg/mL, 9.401 µg/mL, 8.173 µg/mL, and 10.639 µg/mL, respectively. In Sample 3 (late stage), the measurements of these acids were 1999.20 µg/mL, 1501.50 µg/mL, 1079.60 µg/mL, and 1097.60 µg/mL based on an input of 19.992 µg/mL, 15.015 µg/mL, 10.796 µg/mL, and 10.976 µg/mL, respectively. Sample 4 (final stage) yielded the concentrations of acetic acid, propionic acid, butyric acid, and valeric acid, which were 1288.50 µg/mL, 1416.50 µg/mL, 1110.90 µg/mL, and 1162.00 µg/mL, respectively, based on an input of 12.885 µg/mL, 14.165 µg/mL, 11.109 µg/mL, and 11.620 µg/mL, respectively. Overall, these results demonstrate reliable SCFA quantification in standardized human fecal samples.

## 4. Discussion

This study aimed to develop a robust, versatile analytical platform for quantifying SCFAs across various microbiome-related sample types. Given the chemical diversity of SCFAs and the complexity of biological matrices, including pure microbial cultures, low-abundance animal liver, animal fecal samples, and standardized simulated human fecal samples, the optimization of extraction conditions and analytical parameters was essential. The comparative evaluation of headspace-GC and GC-MS/MS techniques identified method-specific advantages and limitations, enabling tailored applications based on sample characteristics. Here, we discuss the critical factors influencing method performance, including solvent selection, derivatization strategies, and matrix-dependent considerations, and highlight the analytical relevance of these optimizations in microbiome-based SCFA research.

The selection of solvent plays a critical role in determining the efficiency of SCFA extraction [23]. Methanol, due to its distinct physicochemical characteristics, surpasses water in extraction effectiveness. As a polar solvent with reduced surface tension, methanol improves the solubilization of fatty acids and augments ester bond cleavage in lipid matrices, thereby enhancing extraction efficiency [24,25]. The lower boiling point and greater volatility of methanol also facilitate straightforward solvent removal, thus minimizing interference during vacuum drying. Accordingly, methanol is considered a more appropriate solvent for SCFA extraction, providing superior efficiency and consistency. However, the selection of the solvent must also align with the requirements of the analytical technique. Although methanol efficiently extracts SCFAs in general, water is preferable for headspace (HS) analysis due to differences in methodology and instrument requirements [26,27]. In HS analysis, analytes must evaporate into the headspace before detection. Water has lower volatility than methanol, which helps provide more stable vaporization and better headspace enrichment [26,27]. The greater volatility of methanol increases the risk of rapid solvent loss and variability in the measured concentrations. Consequently, water is frequently selected as the solvent in HS analysis to improve analyte stability, precision, and reproducibility. In this study, water was used as the extraction solvent for the HS analysis of SCFAs. The procedure was further improved by adding sulfuric acid and sodium chloride to increase sample reactivity [28]. In contrast, for GC-MS/MS analysis, methanol is generally considered more effective for extracting low-volatility compounds such as SCFAs, primarily because of its analytical compatibility. First, methanol’s lower boiling point and higher volatility facilitate the efficient evaporation of volatile analytes upon injection, thus promoting effective column separation. In contrast, the higher boiling point and viscosity of water can lead to extended retention and may compromise the precision of analyte separation [29]. Second, methanol, being a polar solvent, reliably dissolves polar compounds, including fatty acids. For GC-MS/MS analysis, analytes must be vaporized and introduced in the gaseous phase. The use of excessively non-polar solvents may interfere with analyte volatilization and affect analytical performance. The combined polarity and volatility of methanol provide both enhanced solubility and greater analytical reliability [29,30]. Third, when methanol is used during sample preparation, it enables more reproducible and accurate analyses compared with water. Methanol is better suited to typical quantification protocols used in GC-MS/MS assays and reduces complications caused by water’s moisture content. The presence of water can impede ionization, leading to unintended ionization events and analytical artifacts. Conversely, methanol use improves analytical clarity and accuracy. Accordingly, for the extraction and subsequent analysis of short-chain fatty acids using GC-MS/MS, methanol is a more suitable solvent than water [30]. Given methanol’s high volatility, polarity, and overall performance in analytical applications, it yields superior results in this application.

Derivatization is crucial for the effective analysis of short-chain fatty acids (SCFAs), as it increases their volatility, enhances detection sensitivity, and ensures greater analytical precision [31]. The presence of a carboxyl group (-COOH) in SCFAs results in low volatility, presenting significant challenges for direct analysis by gas chromatography (GC) [9,32]. Derivatization, commonly through silylation, which generates derivatives with improved volatility (e.g., —Si(CH_3_)_3_), enables more effective separation and detection by GC [13,33,34]. Furthermore, derivatization increases analytical sensitivity by converting polar molecules into less polar forms, thereby enhancing signal detection in methods such as GC-MS/MS [13,34]. In addition, derivatization facilitates the identification of individual fatty acid species and structures, supporting improved separation and accurate characterization across subclasses of different chain lengths [13,34,35]. In this investigation, MTBSTFA was chosen as the derivatization reagent for the analysis of short-chain fatty acids (SCFAs), owing to its superior chromatographic separation capabilities and its ability to significantly enhance GC sensitivity compared with alternative reagents [34]. MTBSTFA also demonstrates high thermal stability, preserving derivative integrity at elevated temperatures, making it suitable for high-temperature analytical procedures, including GC [34]. Compared with trimethylsilyl (TMS)-based reagents such as BSTFA or MSTFA, MTBSTFA generates tert-butyldimethylsilyl (TBDMS) derivatives, which are generally more chemically stable and less sensitive to moisture [36]. This property is particularly advantageous for biological samples containing residual water or complex matrices, where unstable derivatives may negatively affect reproducibility and quantification accuracy [36]. Previous studies have also reported that MTBSTFA-based derivatization provides improved chromatographic separation and reduced matrix-associated interference for SCFA analysis in complex biological samples [15]. These characteristics provided the basis for selecting MTBSTFA in this study. Methoxyamine hydrochloride (MeOX) was used to stabilize the highly reactive carbonyl groups in certain SCFAs, thereby reducing their reactivity and minimizing side reactions during derivatization. This stabilization step improves analytical accuracy [37]. In summary, due to their low volatility and high polarity, SCFAs require derivatization for reliable analysis by GC-MS/MS [38]. In this study, MeOX and MTBSTFA were co-applied during sample pretreatment. This strategy not only improves the chemical stability and volatility of SCFAs but also enhances thermal stability, thereby reducing unwanted side reactions during measurement. As a result, the developed method demonstrates superior sensitivity and reproducibility for the quantitative determination of SCFAs [37,39]. Earlier studies have demonstrated that acidification and solvent extraction-based methods can achieve acceptable recovery and sensitivity in fecal and plasma samples, particularly when SCFA concentrations are relatively high and matrix complexity is moderate [15,16]. However, these approaches rely on single-quadrupole detection, making them more susceptible to co-eluting compounds and matrix-associated interference. In the current study, these limitations are relevant because the analyzed matrices included animal liver, animal feces, and simulated human fecal samples, which contain diverse endogenous metabolites and impurities. Therefore, a derivatization-based GC-MS/MS workflow was selected to maximize analytical sensitivity and selectivity across different biological matrices. Importantly, the improved analytical performance observed in this study is not attributable solely to tandem MS detection. Rather, it results from the combined effects of extraction, analyte concentration, derivatization, and MS/MS analysis.

To determine the appropriate analytical method for SCFA quantification across different biological sample types (pure microbial cultures, low-abundance animal liver, animal feces, and standardized simulated human fecal samples), the performance of the headspace and GC-MS/MS methods was compared. The reason for selecting these specific sample types is that pure microbial culture samples were used as they contain high SCFA concentrations and low impurities. Low-abundance animal liver samples were included because they contain low SCFA levels and limited sample amounts. Animal fecal samples were selected due to their complex biological impurities and microbial metabolites that may interfere with SCFA detection. Standardized simulated human fecal samples were used to represent highly complex gut-related matrices similar to clinically relevant microbiome samples. These different matrices allowed us to evaluate the analytical methods under various experimental challenges. However, initially, culture medium samples were selected as a model matrix for comparative analysis because the headspace method requires a large sample volume. Our data show the headspace method’s insufficient sensitivity, whereas GC-MS/MS analysis demonstrated high sensitivity and clear detection in diluted samples. Our finding is consistent with previous reports showing that headspace approaches often struggle with low-abundance volatile analytes in diluted matrices. At the same time, tandem mass spectrometry enhances selectivity and lowers detection limits by monitoring specific precursor–product ion transitions [9,40]. Importantly, compared with conventional headspace-GC or single-quadrupole GC-MS methods commonly used in earlier SCFA quantification studies, our GC-MS/MS approach achieved markedly lower detection limits and reduced background noise in diluted samples. Previous studies have reported that headspace-based techniques are prone to signal suppression and reduced sensitivity in complex matrices due to co-eluting volatile compounds and non-selective ionization [12]. In contrast, our tandem MS-based method significantly minimized matrix-derived interference, enabling more accurate peak identification and quantification even in samples containing substantial impurities.

The impact of sample purity and analyte concentration was further assessed. Headspace analysis revealed numerous interfering peaks in undiluted samples likely originating from medium components, dissolved gases, and low-molecular-weight volatiles that co-elute with SCFAs. Similar matrix-derived interferences have been widely reported in headspace analysis, where non-selective ionization and limited chromatographic resolution can obscure precise SCFAs identification [12]. A key improvement demonstrated in the present study is GC-MS/MS’s ability to maintain quantitative reliability across varying levels of matrix complexity and analyte concentration. This aligns with reports showing improved selectivity and quantitative accuracy of tandem MS platforms in metabolite profiling applications [41]. In our study, GC-MS/MS showed approximately 1000-fold higher sensitivity than the headspace method, as GC-MS/MS successfully detected SCFAs at 1 ng/mL, whereas the headspace method failed to identify peaks at the same concentration. Moreover, GC-MS/MS maintained reliable quantification below 100 µg/mL, demonstrating high sensitivity of this approach compared with headspace. In this study, the limits of detection (LOD) and quantification (LOQ) for GC-MS and GC-MS/MS analyses were determined based on signal-to-noise (S/N) ratios of 3 and 10, respectively. However, our study extends these findings by directly validating analytical performance not only in simple culture media but also in low-abundance liver tissues and highly complex fecal matrices within a unified analytical framework.

Furthermore, the analytical performance of these methods was evaluated in complex matrices, demonstrating GC-MS/MS’s ability to selectively detect SCFAs with high precision, even in the presence of impurities. In contrast, headspace analysis produced extensive interference, impeding the identification of target peaks. Regarding this, a study showed the high selectivity and sensitivity of the GC-MS/MS approach in the analysis of complex matrix samples, which aligns with our study [14]. However, our findings further demonstrate that this robustness is maintained in low-concentration biological samples such as liver tissue, where SCFAs are present at trace levels. Likewise, a study reports the ability of this method to quantify microbiota-produced metabolites in different low-abundance sample matrices, including plasma and liver, at different concentrations [42]. Differences in observed SCFAs levels may occur due to several factors, such as biological variability between animal models, dietary conditions, gut microbiota composition, and differences in analytical methods used across studies. Samples with impurities, such as animal fecal samples and standardized simulated human fecal samples, were also analyzed using this approach, demonstrating clear detection of all SCFAs and aligning with the previous report [14,43].

These findings suggest a decision framework to select an appropriate approach (headspace GC-MS and GC-MS/MS) based on sample volume, matrix complexity, expected SCFA concentration, and analytical purpose. Headspace GC-MS provides a rapid and simple workflow without derivatization or drying steps, making it suitable for relatively pure or high SCFA concentration samples, such as microbial culture media. However, its lower sensitivity (1 μg/mL) and susceptibility to matrix interference limit its applicability for complex biological samples. In contrast, GC-MS/MS provides much higher sensitivity (1 ng/mL) and selectivity through derivatization and cleanup procedures, enabling reliable SCFA detection in small-volume and complex samples, including animal liver and fecal samples. Despite the longer sample preparation and higher analytical cost, the GC-MS/MS approach can be a potential choice for low-abundance and heterogeneous biological matrices. Compared with previous SCFA quantification studies that mainly analyzed a single sample type, our study evaluated the method across multiple biological matrices. This demonstrates that the analytical approach can be reliably applied to both simple samples and highly complex biological samples. Such broader validation increases the practical usefulness of the method for future studies investigating microbiome-derived metabolites and host–metabolite interactions.

Headspace analysis eliminates the need for intricate sample pretreatment, enabling rapid acquisition of analytical results [27]. Nevertheless, the absence of pretreatment may allow matrix impurities to interfere with analytical accuracy [27]. In contrast, GC-MS/MS requires extended pretreatment steps, including drying and derivatization [44], resulting in detection as low as 1 ng/mL. The required sample mass is just 0.02 g, making it well suited for the analysis of mouse and rat serum, liver, and feces, as well as simulated human fecal samples containing substantial impurities or when sample volumes are often restricted. However, in this context, a recent clinical trial showed the enrichment of SCFA may improve cirrhosis and hepatic encephalopathy, indicating the importance of proper detection of SCFAs in microbiome-based clinical interventions [45].

There are certain limitations of our study such as a lack of some validation parameters, isotope-labelled or structurally similar internal standards, and extensive statistical analysis. Despite this, we believe our study findings would benefit the selection of an appropriate approach for SCFA analysis. The following summarizes the most appropriate analytical method based on sample concentration, purity, and volume (Table 4). When the sample volume is sufficient, but the concentration is low, GC-MS/MS is preferred for its high sensitivity. For samples with high concentrations, the headspace method is advised because it enables rapid analysis. If the available sample volume is limited, GC-MS/MS is recommended regardless of concentration. When samples exhibit high purity but low concentration, GC-MS/MS is favored for its ability to detect trace levels. In cases of high purity combined with high concentration, the headspace method is most suitable for efficient analysis. For samples with low purity, GC-MS/MS remains appropriate across all concentration levels, as it enables selective derivatization of SCFAs. When the sample volume is large, but the purity is insufficient, GC-MS/MS remains the method of choice. For high-purity samples, the headspace technique offers the advantage of speed. If the sample volume is constrained, GC-MS/MS remains preferable, regardless of purity, as it provides accurate results with minimal sample material.

## 5. Conclusions

In conclusion, this study demonstrates that headspace analysis and GC-MS/MS are complementary approaches for the qualitative and quantitative determination of short-chain fatty acids (SCFAs) in diverse biological specimens. Importantly, the analytical framework established in this study provides practical guidance for choosing suitable SCFA quantification methods based on sample type, matrix complexity, analyte concentration, and sample volume. This approach may support future clinical microbiome studies and targeted metabolomics research by enabling reliable SCFA analysis in complex biological samples, such as fecal and low-abundance tissue samples. In addition, accurate and reproducible SCFA detection may help identify potential biomarkers and improve understanding of microbiome-related diseases and therapeutic effects. Headspace analysis provides rapid screening, whereas GC-MS/MS enables accurate quantification, making both essential techniques for research on human metabolism and the gut microbiota. However, further improvements, including increased sensitivity in headspace analysis and simplified GC-MS/MS protocols, will enhance overall analytical throughput. Ongoing optimization will augment their impact and usage in physiological and clinical investigations.

## Figures and Tables

**Table 1:** **Structure and key characteristics of short-chain fatty acid standards.**

Compound	Acetic Acid	Propionic Acid	Butyric Acid	Valeric Acid
Molecular structure	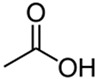	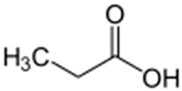	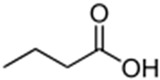	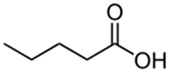
Molecular formula	C_2_H_4_O_2_	C_3_H_6_O_2_	C_3_H_7_COOH	C_5_H_10_O_2_
Molecular weight	60.052 g/mol	74.079 g/mol	88.106 g/mol	102.133 g/mol
CAS No.	64-19-7	79-09-04	107-92-6	109-52-4

This table details the molecular structures, formulas, molecular weights, and CAS (Chemical Abstracts Service) numbers for the standard short-chain fatty acids acetic acid, propionic acid, butyric acid, and valeric acid.

**Table 2:** **Validation data for the headspace GC-MS method in the quantitative analysis of short-chain fatty acids (SCFAs).**

Compound		Acetic Acid	Propionic Acid	Butyric Acid	Valeric Acid
Calibration R^2^		0.995150	0.996924	0.997167	0.995667
Detection properties	Retention Time (min)	3.96	4.80	5.87	7.41
	Peak Detected at 200 μg/mL	Yes	Yes	Yes	Yes
	S/N Ratio (200 μg/mL)	High	High	High	High
	Peak Detected at 1 μg/mL	Weak	Weak	Yes	Yes
	S/N Ratio (1 μg/mL)	Low	Low	Moderate	Moderate
Spike-in recovery (Unit: µg/mL)	Matrix A (Baseline)	266.4507	22.8088	25.6670	30.1633
	Matrix A with 50 μg/mL Spike-in	162.9109	347.0667	304.7817	244.1855
	Recovery Rate (%)	102.96	138.83	121.91	97.67
	Matrix B (Baseline)	251.5533	23.2164	247.3229	30.1503
	Matrix B with 500 μg/mL Spike-in	156.1018	335.5634	315.0746	250.3076
	Recovery Rate (%)	103.53	125.51	118.79	95.46
Selected ion (*m*/*z*)		43, 45	45, 74	60, 73	60, 73

RT, Retention Time; S/N, Signal-to-Noise; R^2^, Coefficient of determination based on calibration curve. Matrix A and Matrix B designate background samples utilized for spike-in recovery evaluation. Spike-in recovery was determined by dividing the measured concentration by the theoretical average of the matrix and the spike, and multiplying by 100. Ion selection relied on characteristic fragment ions (SIM mode) detected by GC-MS.

**Table 3:** **Optimized GC-MS/MS MRM parameters for short-chain fatty acids.**

Compound	Acetic Acid	Propionic Acid	Butyric Acid	Valeric Acid
Calibration R^2^	0.999708	0.999615	0.999591	0.999949
Retention Time (min)	4.398	5.598	6.948	7.248
Selected Ion (*m*/*z*)	75, 117	75, 131	75, 145	73, 147, 189
Ch1 *m*/*z*	117.00 → 75.00	131.00 → 75.10	145.00 → 140.00	147.00 → 73.10
Ch1 CE (V)	9	21	27	15
Ch2 *m*/*z*	118.00 → 76.00	131.00 → 112.00	145.00 → 75.00	189.00 → 147.00
Ch2 CE (V)	6	6	3	3
Ch3 *m*/*z*	117.00 → 71.00	131.00 → 83.00	145.00 → 93.00	148.00 → 60.00
Ch3 CE (V)	12	6	6	36

R^2^, coefficient of determination; RT, retention time; CE, collision energy; *m*/*z*, mass-to-charge ratio; SCFAs, short-chain fatty acids. This table provides optimized multiple reaction monitoring (MRM) parameters for quantifying four representative SCFAs—acetic acid, propionic acid, butyric acid, and valeric acid—using GC-MS/MS. Each SCFA was monitored by three precursor-to-product ion transitions (Ch1–Ch3), selected based on product ion scans and their respective signal intensities. The optimal collision energy (CE) was established for each transition to maximize detection sensitivity. Selected ions correspond to the dominant precursor ions used for transition setup. Calibration R^2^ values indicate the linearity of calibration curves using *n* ≥ 5 concentrations.

**Table 4:** **Comparison of Headspace and GC-MS/MS Analytical Methods.**

Category	Headspace GC-MS	GC-MS/MS
Sample preparation time	Short, as neither derivatization nor drying steps are necessary	Long, as both drying and derivatization steps are involved
Detection limit	1 μg/mL	1 ng/mL
Sample purity requirement	Recommended for use with clean samples because no preprocessing is performed	Can be applied to impure samples owing to derivatization and cleanup procedures
Required sample volume	At least 5 mL	At least 0.02 g
Eligible sample categories	Samples with high analyte concentrations, particularly pure microbial cultures	Samples with low analyte concentrations and complex matrices, including low-abundance animal liver, animal feces, and standardized simulated human fecal samples

## Data Availability

The data supporting this study are available from the corresponding author upon reasonable request.

## Supplementary Materials

The following supporting information can be downloaded at https://www.mdpi.com/article/10.3390/life16060974/s1, Table S1. Operating conditions for headspace GC-MS analysis of short-chain fatty acids; Table S2. Instrumental conditions used for quantitative analysis of SCFAs by GC-MS/MS; Table S3. Summary of Short-Chain Fatty Acid (SCFA) Quantification in Various Sample Types Using the GC-MS/MS Method; Figure S1. Sample preparation and instrumentation setup for headspace GC-MS analysis; Figure S2. Instrumental condition settings on the headspace sampler control panel; Figure S3. GC method parameters configured for headspace analysis; Figure S4. Ion method configuration for headspace GC-MS analysis; Figure S5. Analytical sequence setup for headspace GC-MS analysis; Figure S6. Workflow for sample pretreatment and derivatization prior to GC-MS/MS analysis of SCFAs; Figure S7. Method configuration for GC-MS/MS analysis; Figure S8. Sample injection sequence setup for GC-MS/MS analysis; Figure S9. Calibration curves of short-chain fatty acids (SCFAs) obtained using headspace GC-MS; Figure S10. Representative headspace GC-MS chromatograms of SCFA standard mixtures at different concentrations; Figure S11. Characteristic fragment ions (*m*/*z*) of short-chain fatty acids (SCFAs) detected by headspace GC-MS; Figure S12. Calibration curves of short-chain fatty acids (SCFAs) obtained using GC-MS/MS; Figure S13. Representative GC-MS/MS chromatogram of SCFAs at 1 ng/mL; Figure S14. MS table view displaying the retention windows and expected peak elution times for SCFAs analyzed by GC-MS/MS; Figure S15. Representative fragment ion spectra (*m*/*z*) of short-chain fatty acids (SCFAs) identified by GC-MS/MS; Figure S16. Setup for SCFA product ion scans utilizing the SmartDatabase in GC-MS/MS; Figure S17. Collision energy (CE) optimization for individual SCFA transitions in GC-MS/MS quantification; Figure S18. Summary of optimal collision energies (CEs) and product ion intensities for each SCFA transition in GC-MS/MS analysis; Figure S19. Quantification of SCFAs in Culture Media Samples Using the Headspace Method.
