# Supplementary material for: Development of Mass Spectrometry-Based SCFA Analysis Methods in Diverse Samples for Microbiome Research
Source: Life (Basel). 2026 Jun 9;16(6):974. doi: 10.3390/life16060974 (PMC13302153; doi:10.3390/life16060974)
Supplement: Supplementary file 1 [file life-16-00974-s001.zip › life-4313988-supplementary.pdf]

life

MDPI

Supplementary Materials

Experimental parameter		Instrumental condition	
GC	Instrument	Perkin Elmer Clarus 690 GC, Clarus SQ8-GC-MS, TurboMatrix Headspace system	
	Column	Elite-FFAP (30 m * 0.25 mm, 0.25 um)	
	Injection Temp.	250 ℃	
	Carrier Gas	He	
	Flow	1.00 mL/min	
	Oven program	120 ℃→ 5℃/min → 200 ℃	
MS	Ionization Mode	Ei+	
	Ion source Temp.	250 ℃	
	SIM ions (m/z)	Acetic acid	43, 45
		Propionic acid	45, 74
		Butyric acid	60, 73
		Valeric acid	60, 73

Table S1. Operating conditions for headspace GC-MS analysis of short-chain fatty acids.

A summary of instrumental conditions for GC-MS analysis with headspace sampling is presented. The system consists of a PerkinElmer Clarus 690 GC connected to a Clarus SQ8-MS and TurboMatrix headspace sampler. Separation is performed on an Elite-FFAP capillary column (30 m × 0.25 mm, 0.25 μm film thickness) using helium as the carrier gas at a flow rate of 1.00 mL/min. The oven program is set from 120 °C to 200 °C at 5 °C/min. Mass spectrometric analysis is performed in electron ionization (EI+) mode at 250 °C ion source temperature, with selected ion monitoring (SIM) for the detection of acetic acid (m/z 43, 45), propionic acid (m/z 45, 74), butyric acid (m/z 60, 73), and valeric acid (m/z 60, 73).

Parameter		Instrumental condition
GC	Instrument	SHIMADZU – GC: 2010 Plus, GC-MS: TQ8040
	Column	DB-5MS (30 m * 0.25 mm, 0.25 um)
	Injection Temp.	250 ℃
	Carrier Gas	He
	Flow	1.00 mL/min
	Injection Mode	Split 5:1
	Injection Volume	1 μL
	Oven program	60 ℃ (1min) → 10℃/min → 200 ℃ → 30℃/min → 325℃(5min)
MS/MS	Transfer line temperature	290 ℃
	Ion source temperature	230 ℃

	Quantification ion	Acetic acid	117>75, 118>76, 117>71
		Propionic acid	131>75, 131>112, 131>83
		Butyric acid	145>140, 145>75, 145>93
		Valeric acid	147>73, 189>147, 148>60

Table S2. Instrumental conditions used for quantitative analysis of SCFAs by GC-MS/MS. This table summarizes the comprehensive GC and MS/MS operational parameters used for the quantitative determination of short-chain fatty acids (SCFAs). The GC-2010 Plus system (Shimadzu) was configured with a DB-5MS column (30 m × 0.25 mm, 0.25 μm), utilizing an injection temperature of 250 °C in split mode (5:1) with a 1 μL sample volume. The oven program initiated at 60 °C for 1 min, followed by a ramp to 200 °C at 10 °C/min, then further to 325 °C at 30 °C/min, with a final hold at 325 °C for 5 min. Helium served as the carrier gas at a constant flow of 1.00 mL/min. The transfer line and ion source were maintained at 290 °C and 230 °C, respectively. Quantification relied on multiple reaction monitoring (MRM) mode, incorporating optimized precursor-product ion transitions for each SCFA: acetic acid (117→75, 118→76, 117→71), propionic acid (131→75, 131→112, 131→83), butyric acid (145→140, 145→75, 145→93), and valeric acid (147→73, 189→147, 148→60).

Table S3. Summary of Short-Chain Fatty Acid (SCFA) Quantification in Various Sample Types Using the GC-MS/MS Method**.**

Samples type	Sample No.	Analyte	Amount (μg/mL)	Result (μg/mL)
Pure microbial cultures	Sample 1	Acetic acid	1.527	152.7
		Propionic acid	0.557	55.7
		Butyric acid	0.013	1.3
		Valeric acid	1.599	159.9
	Sample 2	Acetic acid	1.728	172.8
		Propionic acid	0.595	59.5
		Butyric acid	0.015	1.5
		Valeric acid	1.026	102.6
	Sample 3	Acetic acid	1.654	165.4
		Propionic acid	0.561	56.1
		Butyric acid	0.013	1.3
		Valeric acid	0.996	99.6
	Sample 4	Acetic acid	1.588	158.8
		Propionic acid	0.526	52.6
		Butyric acid	0.013	1.3
		Valeric acid	0.979	97.9
Low-abundance mice liver	Sample 1	Acetic acid	16.556	1655.6
		Propionic acid	4.232	423.2
		Butyric acid	3.877	387.7
		Valeric acid	16.231	1623.1
	Sample 2	Acetic acid	50.148	5014.80
		Propionic acid	3.094	309.40
		Butyric acid	1.611	161.10
		Valeric acid	15.954	1595.4

	Sample 3	Acetic acid	20.076	2007.60
		Propionic acid	3.012	301.20
		Butyric acid	11.618	1161.80
		Valeric acid	15.931	1593.90
	Sample 4	Acetic acid	56.939	5693.9
		Propionic acid	1.603	160.30
		Butyric acid	0.849	84.90
		Valeric acid	16.150	1615.00
Animal fecal samples	Sample 1	Acetic acid	2.200	220.00
		Propionic acid	0.268	26.80
		Butyric acid	0.078	7.80
		Valeric acid	3.506	350.60
	Sample 2	Acetic acid	1.083	108.30
		Propionic acid	0.201	20.10
		Butyric acid	0.060	6.00
		Valeric acid	3.457	345.70
	Sample 3	Acetic acid	1.252	125.2
		Propionic acid	0.153	15.30
		Butyric acid	0.094	9.40
		Valeric acid	3.784	378.4
	Sample 4	Acetic acid	0.852	85.20
		Propionic acid	0.221	22.10
		Butyric acid	0.053	5.30
		Valeric acid	3.880	388.00
Standardized simulated human fecal samples	Sample 1	Acetic acid	10.640	1064.00
		Propionic acid	10.318	1031.80
		Butyric acid	7.735	773.50
		Valeric acid	10.775	1077.50
	Sample 2	Acetic acid	11.800	1180.00
		Propionic acid	9.401	940.10
		Butyric acid	8.173	817.30
		Valeric acid	10.639	1063.90
	Sample 3	Acetic acid	19.992	1999.20
		Propionic acid	15.015	1501.50
		Butyric acid	10.796	1079.60
		Valeric acid	10.976	1097.60
	Sample 4	Acetic acid	12.885	1288.50
		Propionic acid	14.165	1416.50
		Butyric acid	11.109	1110.90
		Valeric acid	11.620	1162.00

Samples 1–4 correspond to the left-to-right order shown in Figure 2.

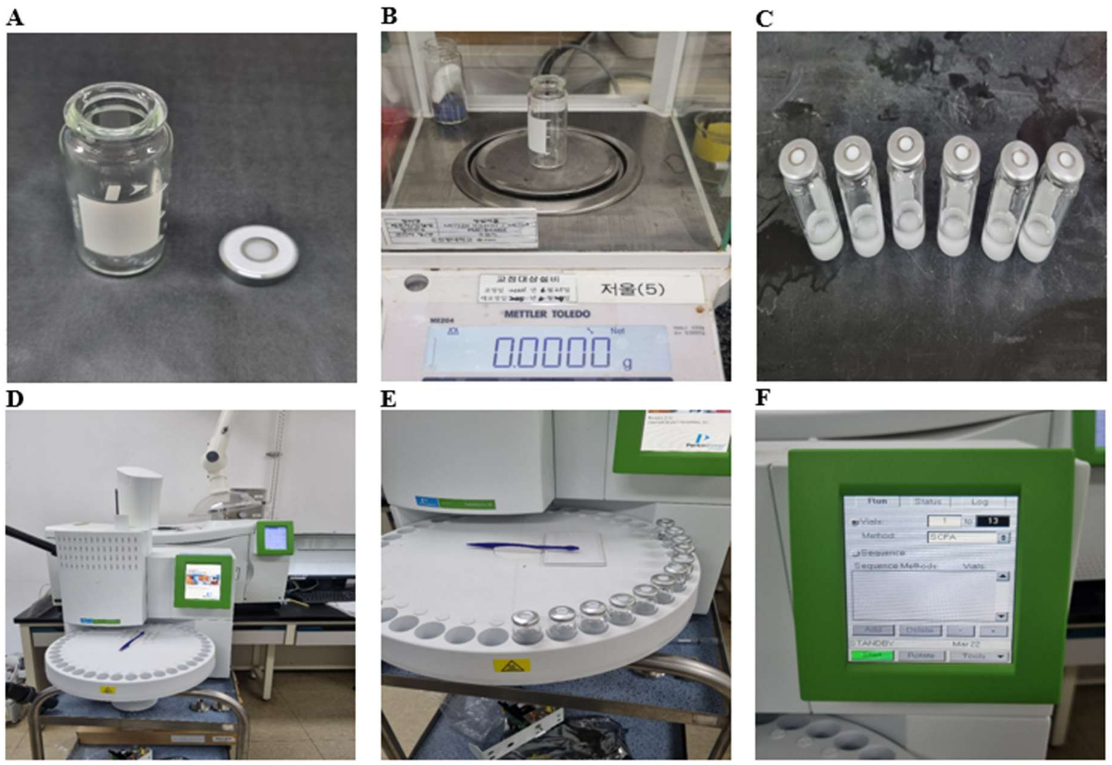

Figure S1. Sample preparation and instrumentation setup for headspace GC-MS analysis. (A) Prepare a headspace vial and secure it with a PTFE-lined crimp cap for volatile compound detection, (B) weigh the empty vial using an analytical balance for precise sample measurement, and (C) introduce the prepared solution containing sodium chloride and sulfuric acid before sealing the vial. (D) Load the capped vials into the autosampler tray of the headspace-GC-MS platform, (E) organize them according to the analytical sequence for automation, and (F) initiate the process by selecting the required method and pressing START on the system control panel. This standardized protocol promotes reliable and reproducible measurement of volatile fatty acids using headspace gas chromatography–mass spectrometry.

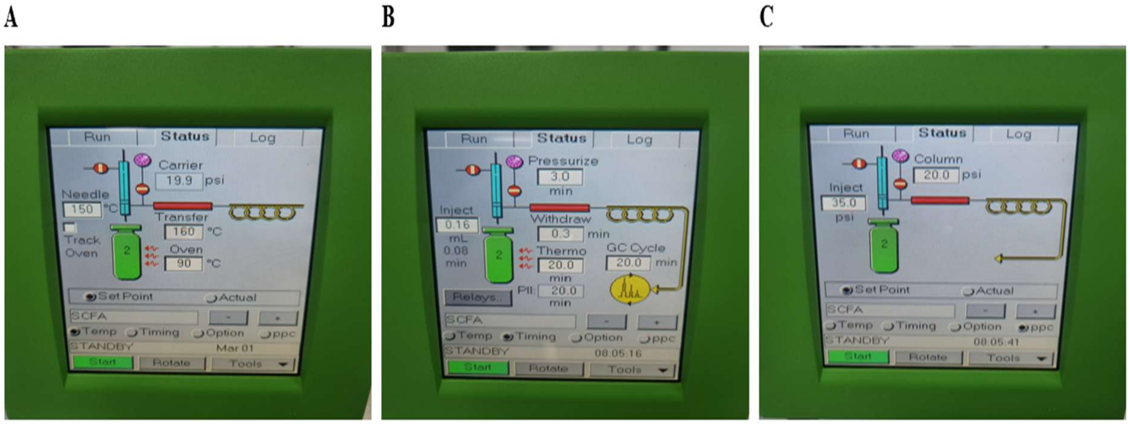

Figure S2. Instrumental condition settings on the headspace sampler control panel. (A) Set the temperatures for the needle, transfer line, and oven to 150 °C, 160 °C, and 90 °C, respectively. (B) Specify the timing parameters, including a pressurization time of 3.0 min, injection volume of 0.16 mL, withdrawal time of 0.3 min, thermostat time of 20.0 min, and total GC cycle duration of 20.0 min. (C) Configure the pneumatic pressure control (PPC) with the injection pressure at 35.0 psi and column pressure at 20.0 psi. These optimized conditions facilitate precise thermal equilibration, consistent sample transfer, and robust headspace GC-MS data acquisition.

https://doi.org/10.3390/xxxxx

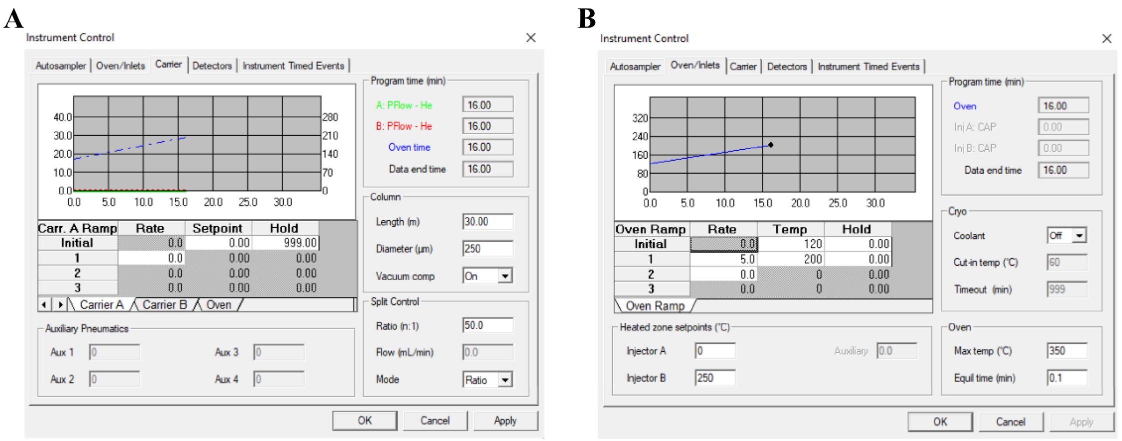

Figure S3. GC method parameters configured for headspace analysis. (A) The oven temperature program for the GC starts at 120 °C, ramps at 5 °C/min to a final temperature of 200 °C, with a total runtime of 16 minutes. The injector is held at 250 °C to promote effective vaporization of analytes. (B) Helium is used as the carrier gas, with a split ratio of 50:1 and a flow rate of 1.00 mL/min. A 30 m × 0.25 mm, 0.25 µm film-thickness capillary column is employed to achieve highly reproducible and efficient separation of volatile fatty acids by headspace GC-MS.

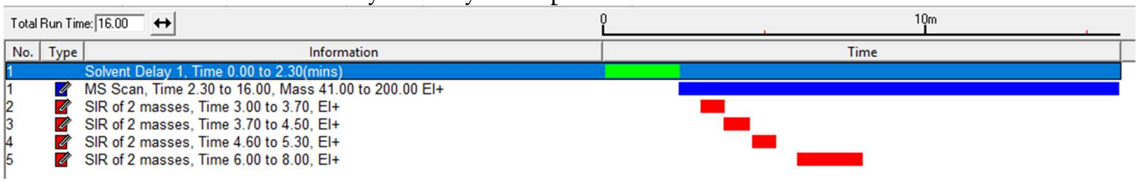

Figure S4. Ion method configuration for headspace GC-MS analysis. To avoid background noise from registering before analyte elution, a solvent delay of 2.3 minutes is applied before MS data collection. The mass spectrometer operates in selected ion recording (SIR) mode, targeting each analyte within its specific retention time window for optimal detection of short-chain fatty acids. This ion method configuration enables selective, quantitatively reliable measurements within the defined retention times.

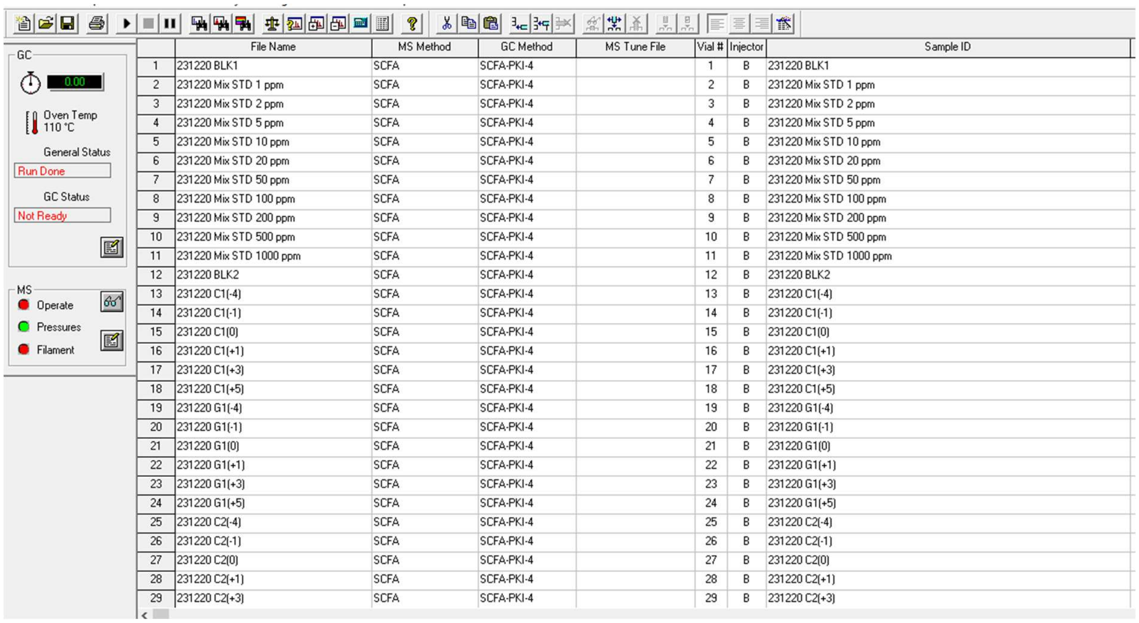

Figure S5. Analytical sequence setup for headspace GC-MS analysis. To ensure instrument stabilization prior to analysis, the sequence begins with the injection of base (blank) vials, followed by standard solutions in ascending concentration to generate the calibration curve. Base vials are

reinjected after calibration to monitor and prevent contamination, and then biological samples are analyzed as prescribed. The MS parameters are set to predefined ion values for selected ion recording (SIR), and GC conditions are configured using validated oven and flow parameters. This analytical workflow is designed to deliver consistent quantification and reduce variability across measurements.

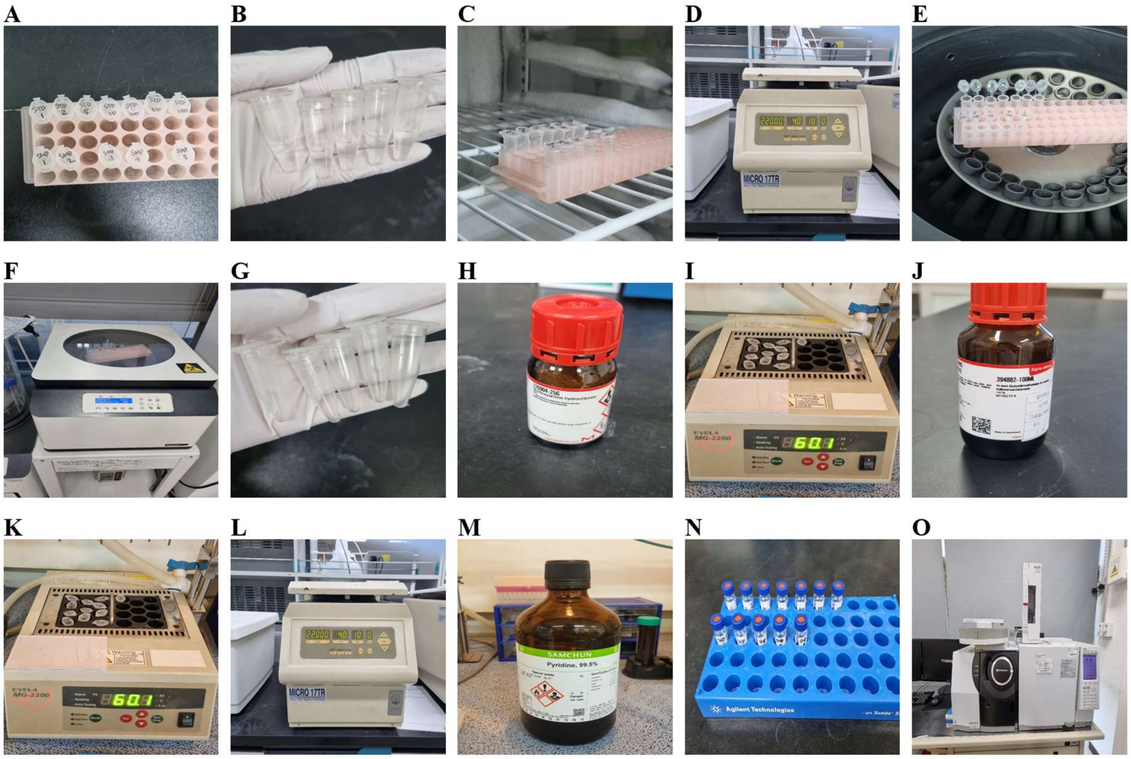

Figure S6. Workflow for sample pretreatment and derivatization prior to GC-MS/MS analysis of SCFAs. This figure details each step of the sample preparation protocol employed for the quantitative determination of short-chain fatty acids (SCFAs) by GC-MS/MS. (A) Standard solutions and biological samples were initially dispensed into Eppendorf tubes. (B) Subsequently, 0.1 M NaOH and methanol were added to each tube. (C) After thorough mixing, the tubes were incubated at –20 °C for 20 minutes. (D) The processed samples were then centrifuged at 22,000 × g for 10 minutes at 4 °C. (E) Supernatants obtained post-centrifugation were transferred into vacuum concentrator tubes, (F) which were tightly sealed, and (G) the samples were subjected to complete drying under reduced pressure. (H) Methoxyamine hydrochloride (MeOX) was dissolved in pyridine to prepare the derivatization reagent. (I) This reagent was added to the dried samples, followed by incubation at 60 °C for 90 minutes to stabilize reactive carbonyl groups. (J) MTBSTFA was subsequently introduced as the derivatizing agent, and (K) the mixture was incubated again at 60 °C for 30 minutes. (L) Following derivatization, a secondary centrifugation step was performed to remove any residual particulates. (M) Pyridine was freshly prepared for subsequent use, and (N) 70 μL of the resulting supernatant was combined with 140 μL of pyridine prior to transfer into autosampler vials. (O) The prepared, derivatized samples were ultimately injected into the GC-MS/MS instrument for analysis.

A

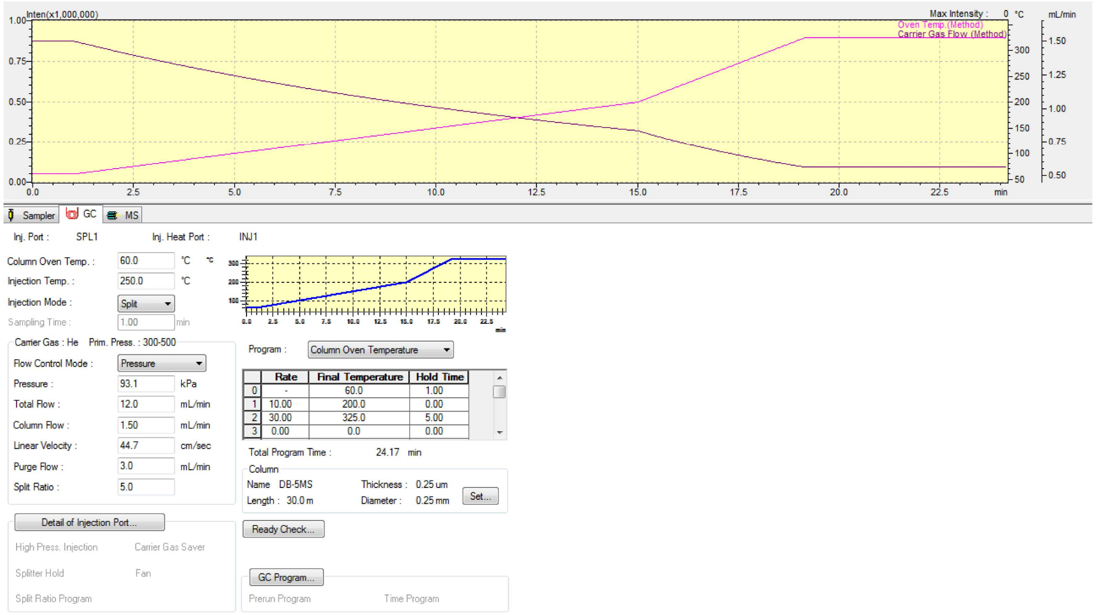

B

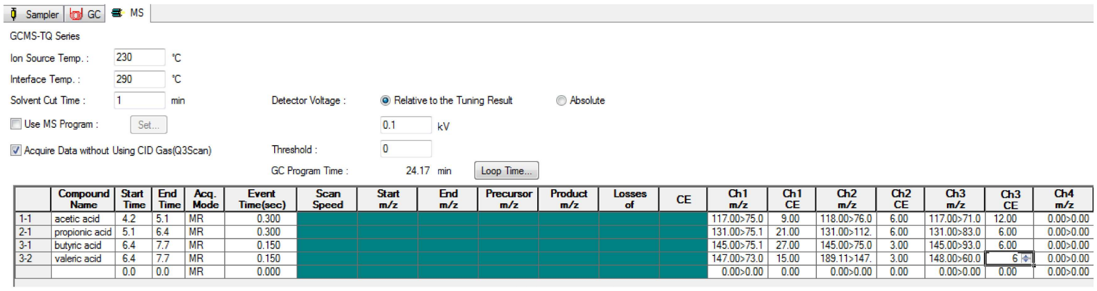

Figure S7. Method configuration for GC-MS/MS analysis. This figure outlines the instrumental configuration and analytical settings established for targeted detection of short-chain fatty acids (SCFAs) via GC-MS/MS. (A) The GC oven temperature program used for SCFA analysis is depicted. The oven temperature was initially set to 60 °C for 1 minute, then increased to 200 °C at 10 °C/min, then further ramped to 325 °C at 30 °C/min, and maintained for 5 minutes. Utilization of this gradient facilitated optimal separation and resolution of all target analytes. (B) MRM (Multiple Reaction Monitoring) settings employed for SCFA quantification in the TQ8040 triple quadrupole mass spectrometer. Optimized precursor and product ion transitions, along with corresponding collision energies (CE), were specified for each analyte—acetic acid, propionic acid, butyric acid, and valeric acid—to ensure high sensitivity and selectivity during detection.

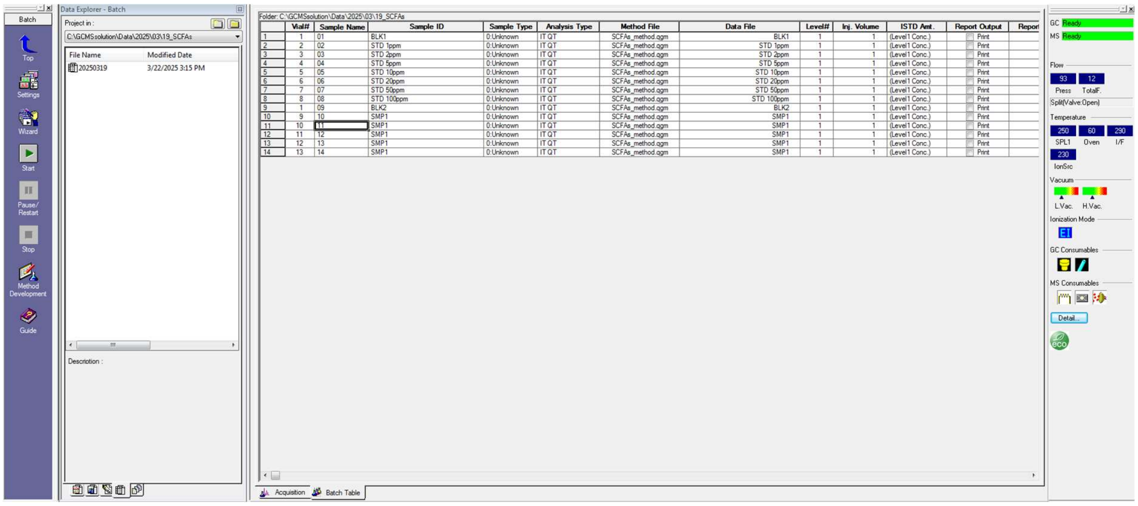

Figure S8. Sample injection sequence setup for GC-MS/MS analysis. A systematic injection sequence was established to achieve precise quantification of short-chain fatty acids (SCFAs) and to mitigate carryover during GC-MS/MS analysis effectively. The sequence commenced with injections of base vials to condition and stabilize the instrument, immediately followed by standard solutions at incrementally increasing concentrations for calibration curve construction. Prior to the introduction of each new set of biological samples, a base vial was injected to reduce the risk of contamination originating from preceding sample analyses. This purposeful alternation between base vials and sample injections was implemented to enhance system stability and sustain analytical robustness. The pre-specified method file encompassed all relevant ion transitions and instrument operating parameters for targeted MRM data acquisition.

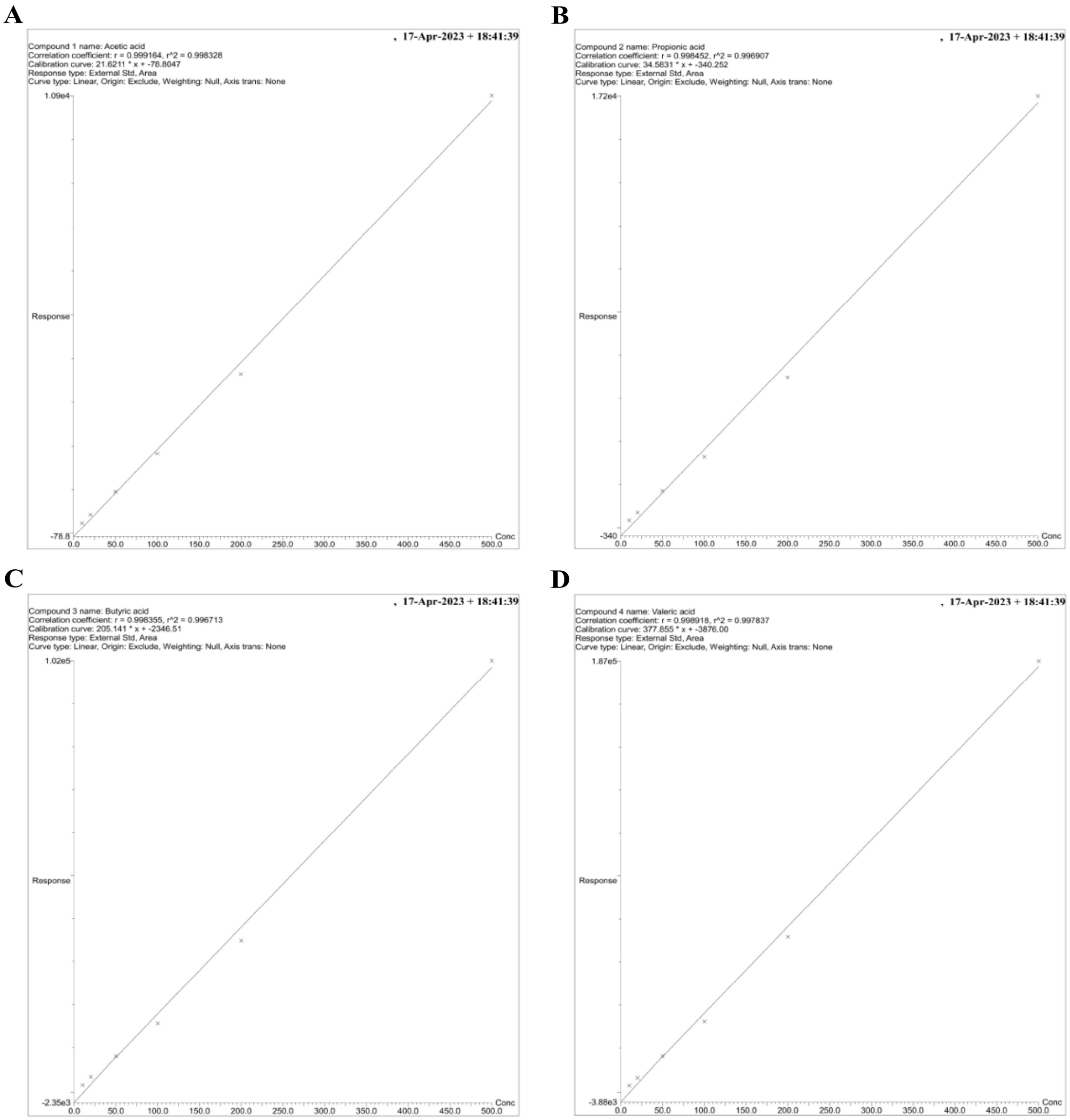

Figure S9. Calibration curves of short-chain fatty acids (SCFAs) obtained using headspace GC-MS. The calibration curves illustrate the linear relationship between detector response and concentration for each SCFA analyzed by headspace GC-MS within the tested ranges. (A) Acetic acid achieved an R^2 of 0.998328, signifying a robust linear correlation across its calibration interval. (B) Propionic acid displayed an R^2 value of 0.996907. (C) Butyric acid showed high linearity with an R^2 of 0.996713. (D) Valeric acid also exhibited a strong linear response with an R^2 value of 0.997837. These findings support the method's reliability for the quantitative determination of SCFAs utilizing headspace detection.

A

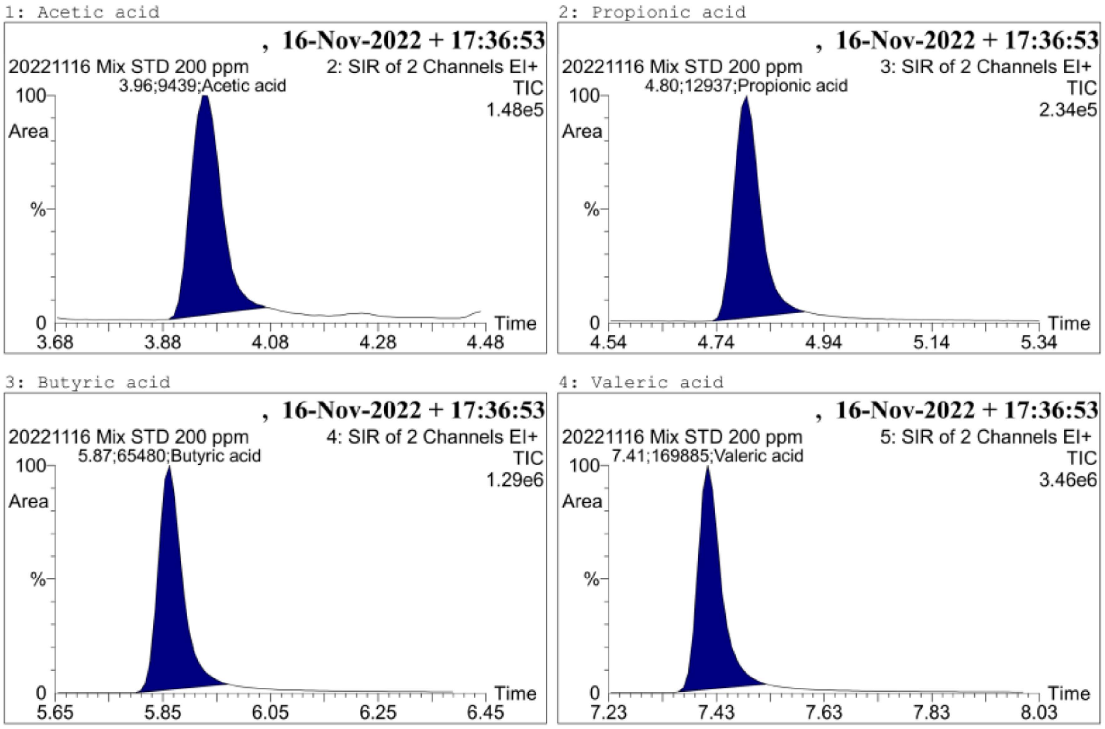

B

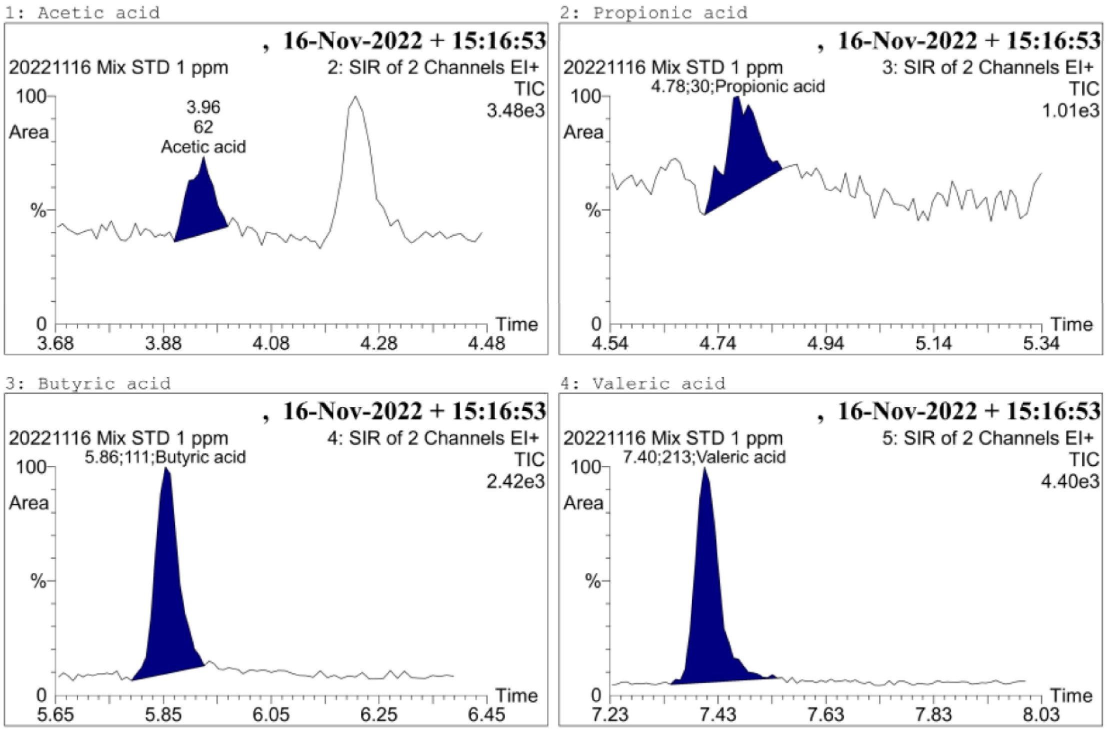

Figure S10. Representative headspace GC-MS chromatograms of SCFA standard mixtures at different concentrations. This figure compares chromatographic profiles of four short-chain fatty acids (SCFAs)—acetic acid, propionic acid, butyric acid, and valeric acid—analyzed by headspace GC-MS at two concentrations. (A) At 200 μg/mL, prominent and distinct peaks with high signal intensity were observed for all SCFAs. (B) At 1 μg/mL, butyric acid and valeric acid remained detectable with adequate sensitivity. In contrast, acetic acid and propionic acid exhibited diminished signals or low signal-to-noise ratios,

indicating reduced detectability at lower concentrations. Consequently, 1 μg/mL was determined as the lowest concentration at which simultaneous headspace-based detection could be reliably achieved.

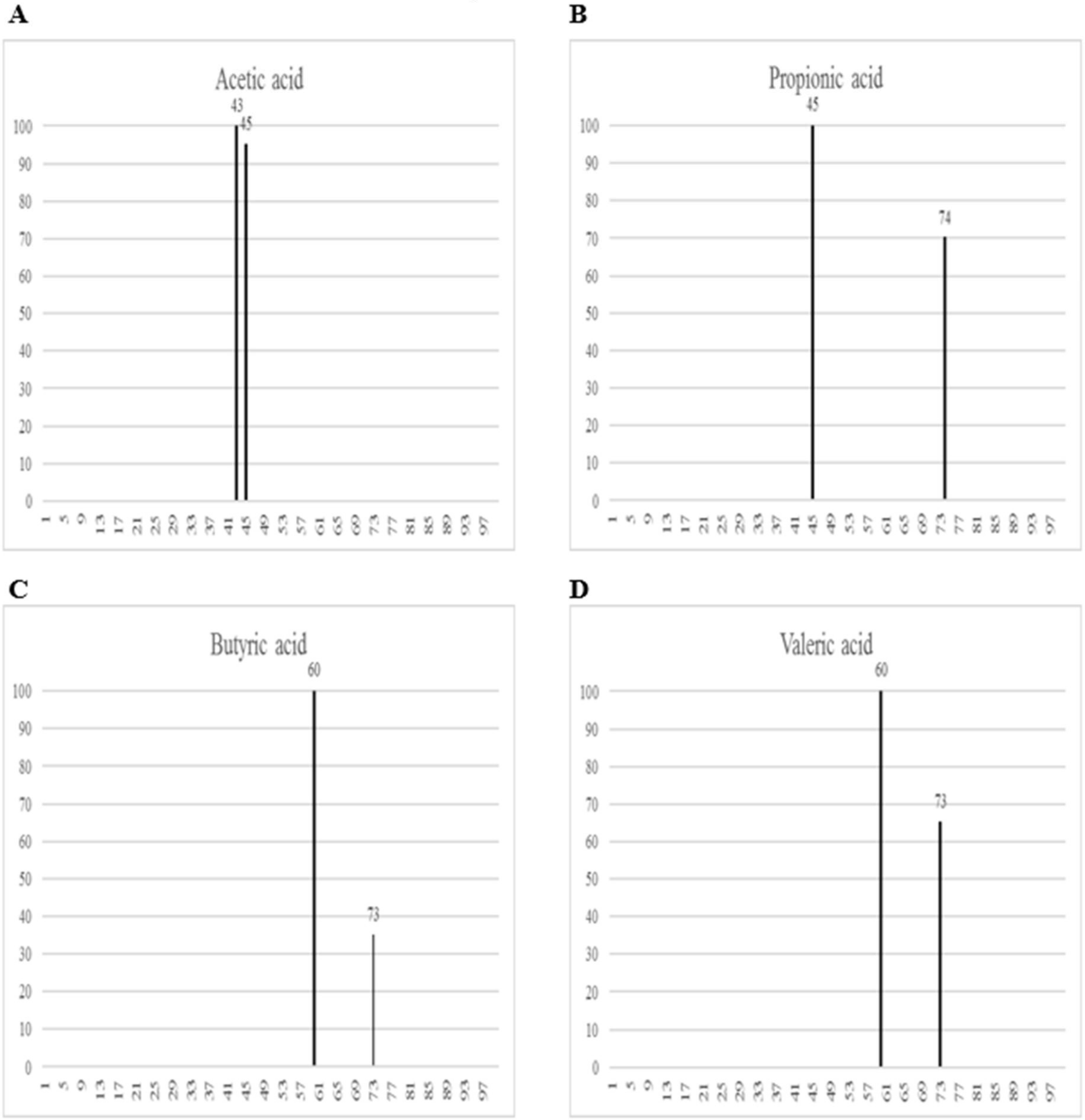

Figure S11. Characteristic fragment ions (m/z) of short-chain fatty acids (SCFAs) detected by headspace GC-MS. This figure illustrates the selected-ion monitoring (SIM) spectra used to distinguish each SCFA in headspace GC-MS, facilitating their identification. (A) Acetic acid was identified using fragment ions at m/z 43 and 45. (B) Ions at m/z 45 and 74 characterized propionic acid. (C) Butyric acid was mainly detected as fragment ions at m/z 60 and 73. (D) Valeric acid was also monitored using fragment ions at m/z 60 and 73. These ion transitions enabled specific detection and enhanced confidence in the identification of each compound within complex matrices.

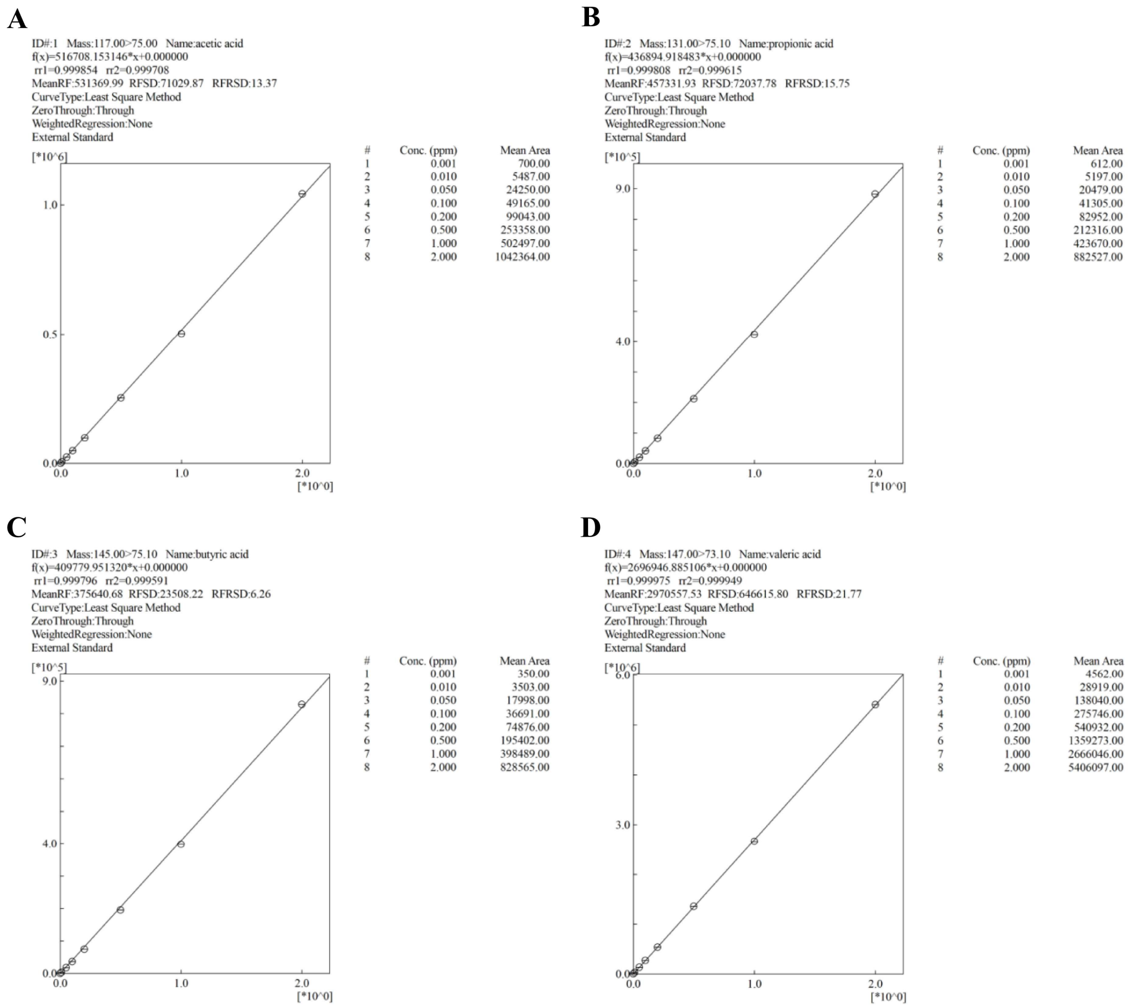

Figure S12. Calibration curves of short-chain fatty acids (SCFAs) obtained using GC-MS/MS. The detector's linearity for quantifying SCFAs was assessed using multiple reaction monitoring (MRM) mode by GC-MS/MS. (A) Acetic acid yielded an R^2 value of 0.999708. (B) Propionic acid exhibited an R^2 value of 0.999615, confirming its linear response. (C) Butyric acid achieved an R^2 of 0.999591. (D) Valeric acid displayed the strongest correlation, reaching an R^2 of 0.999949. Across the concentration range tested (1 ng/mL–2 μg/mL), all analytes exhibited highly linear responses, confirming the method's reliability for precise and accurate quantification of SCFAs.

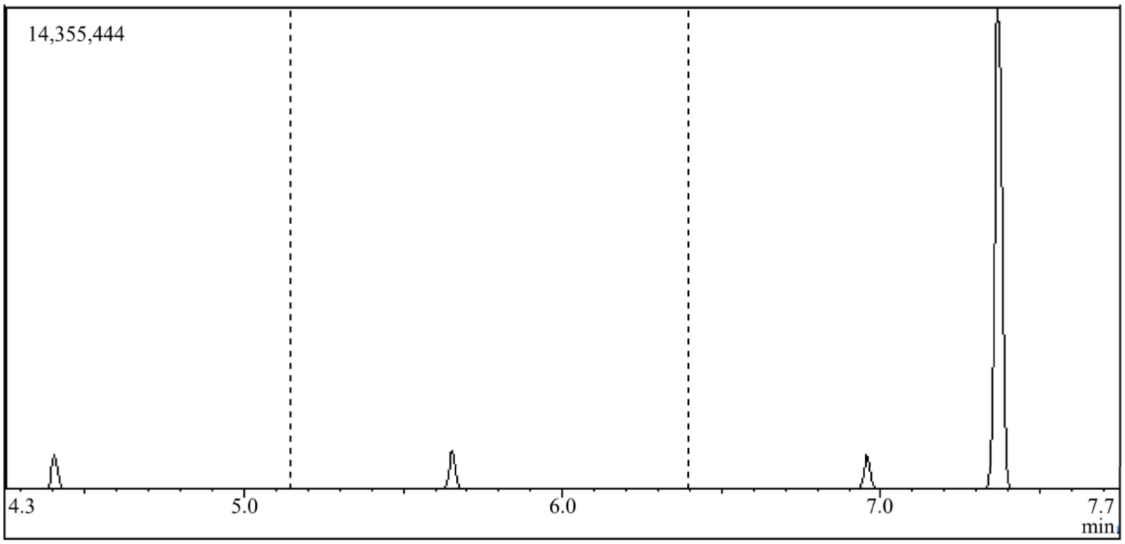

Figure S13. Representative GC-MS/MS chromatogram of SCFAs at 1 ng/mL. The chromatogram shows the separation of a mixture containing four SCFAs at 1 ng/mL using GC-MS/MS under multiple reaction monitoring (MRM) conditions. From left to right, acetic acid, propionic acid, butyric acid, and valeric acid were distinctly resolved. Detection of all SCFAs with clear retention times and excellent baseline separation underscores the method's high sensitivity and appropriateness for low-level quantification of SCFAs.

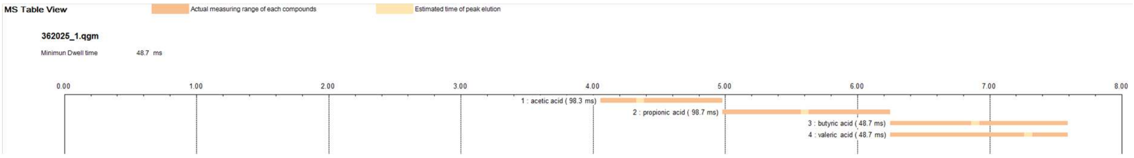

Figure S14. MS table view displaying the retention windows and expected peak elution times for SCFAs analyzed by GC-MS/MS. Retention time intervals and anticipated elution points for each analyte were established during GC-MS/MS runs. Acetic acid exhibited a retention window of approximately 4 to 5 min, with the maximum peak at 4.398 min. For propionic acid, the retention time ranged from 5 to 6.2 min, with the main peak at 5.598 min. Butyric acid was observed within a range of 6.2 to 7.6 min, peaking at 6.948 min. Valeric acid shared this interval (6.2–7.6 min), with its peak elution occurring at 7.248 min.

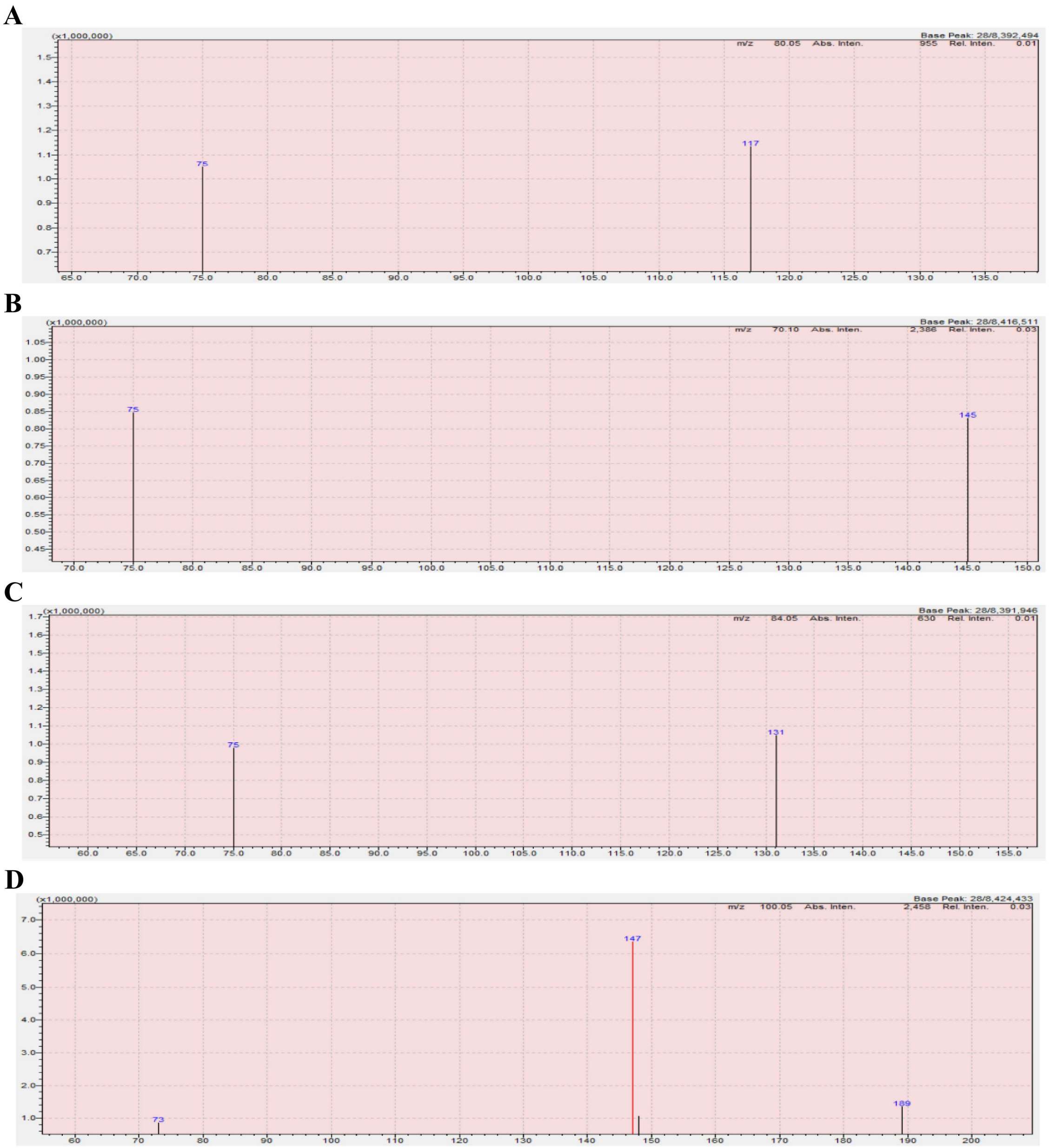

Figure S15. Representative fragment ion spectra (m/z) of short-chain fatty acids (SCFAs) identified by GC-MS/MS. Each panel presents signature ion fragments that facilitate precise, compound-specific identification of SCFAs in GC-MS/MS analyses. (A) Acetic acid was detected using transitions at m/z 75 and 117. (B) Transitions at m/z 75 and 131 were characteristic of propionic acid. (C) Butyric acid was assigned transitions at m/z 75 and 145. (D) Valeric acid detection relied on three transitions: m/z 73, 147, and 189. These transitions were chosen for their analytical reproducibility and robust signal intensities for multiple reaction monitoring (MRM)-based quantification.

No.	File Name
1	CE03V_TargetComp_ProductionScan.qgd
2	CE06V_TargetComp_ProductionScan.qgd
3	CE09V_TargetComp_ProductionScan.qgd
4	CE12V_TargetComp_ProductionScan.qgd
5	CE15V_TargetComp_ProductionScan.qgd
6	CE18V_TargetComp_ProductionScan.qgd
7	CE21V_TargetComp_ProductionScan.qgd
8	CE24V_TargetComp_ProductionScan.qgd
9	CE27V_TargetComp_ProductionScan.qgd
10	CE30V_TargetComp_ProductionScan.qgd
11	CE33V_TargetComp_ProductionScan.qgd
12	CE36V_TargetComp_ProductionScan.qgd
13	CE39V_TargetComp_ProductionScan.qgd
14	CE42V_TargetComp_ProductionScan.qgd
15	CE45V_TargetComp_ProductionScan.qgd

No.	Compound Name	Ret. Time	Ret. Index	Precursor Ion1	Event1	Precursor Ion2	Event2	Precursor Ion3	Event3
1	acetic acid	4.353	0	117.000	1	75.000	2	118.000	3
2	propionic acid	5.592	0	131.000	1	75.000	2	73.000	3
3	butyric acid	6.874	0	75.000	1	145.000	2	73.000	3
4	valeric acid	7.288	0	147.000	4	189.000	5	148.000	6

Figure S16. Set up for SCFA product ion scans utilizing the SmartDatabase in GC-MS/MS. The optimal collision energy (CE) for each precursor-to-product ion transition was determined for acetic acid, propionic acid, butyric acid, and valeric acid by developing a product-ion scan protocol using the SmartDatabase function. CE settings were systematically tested in increments of 3 from 3 to 45. Three precursor ions were selected for each analyte, with up to three CE settings per ion to assess fragmentation performance. Retention time and transition parameters for each analyte are provided to facilitate subsequent MRM optimization and method verification.

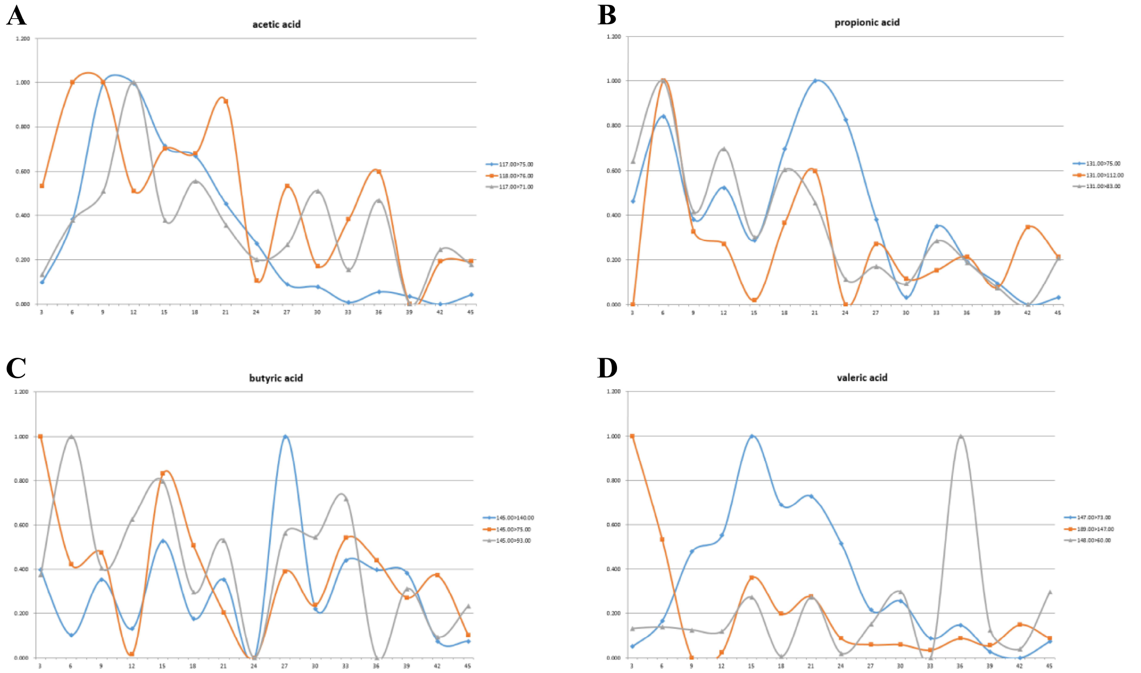

Figure S17. Collision energy (CE) optimization for individual SCFA transitions in GC-MS/MS quantification. Optimal CE values for each MRM transition were established by analyzing product ion intensities from acetic acid, propionic acid, butyric acid, and valeric acid over a range of collision energies. The data were normalized and plotted as a function of the CE range from 3 to 45 eV. (A) Acetic acid demonstrated distinct CE maxima across three monitored transitions. (B) Propionic acid reached maximal intensities at unique CE values across transitions. (C) Butyric acid displayed characteristic, though variable, CE-dependent maxima for each transition. (D) Valeric acid showed marked signal enhancement under specific CE conditions. These experimental findings informed the selection of final MRM parameters utilized for SCFA quantitation.

A

Product m/z	117.00>75.00	118.00>76.00	117.00>71.00
Max Int.	250	63	60
CE	9	6	12

B

Product m/z	131.00>75.00	131.00>112.00	131.00>83.00
Max Int.	77	70	68
CE	21	6	6

C

Product m/z	145.00>140.00	145.00>75.00	145.00>93.00
Max Int.	85	74	73
CE	27	3	6

D

Product m/z	147.00>73.00	189.00>147.00	148.00>60.00
Max Int.	315	297	158
CE	15	3	36

Figure S18. Summary of optimal collision energies (CEs) and product ion intensities for each SCFA transition in GC-MS/MS analysis. To identify the most suitable collision energies for each MRM transition, product ion intensities were systematically evaluated over a range of CE values, and the maximal intensities were documented. (A) Acetic acid transitions (117→75, 118→76, 117→71) exhibited the highest intensities at CE values of 9, 6, and 12 eV, respectively. (B) Propionic acid transitions (131→75, 131→112, 131→83) provided optimal responses at 21, 6, and 6 eV, respectively. (C) Butyric acid transitions (145→140, 145→75, 145→93) reached maximum intensities at CE values of 27, 3, and 6 eV, respectively. (D) Valeric acid transitions (147→73, 189→147, 148→60) reached their strongest signals at 15, 3, and 36 eV. These results informed the selection of final MRM parameters for quantitative analyses.

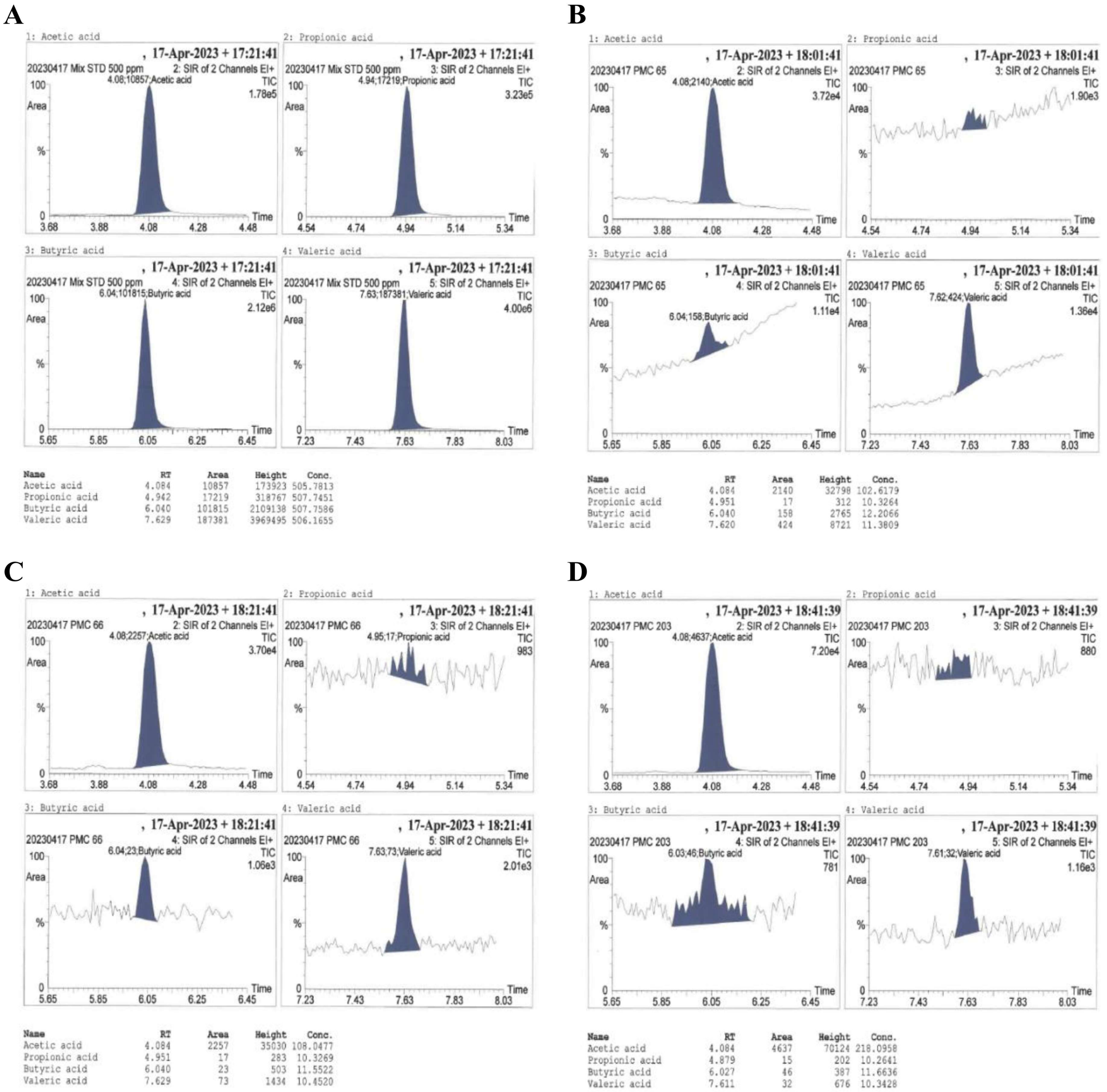

A

Name	RT	Area	Height	Conc.
Acetic acid	4.084	10857	173923	505.7813
Propionic acid	4.942	17219	318767	507.7451
Butyric acid	6.040	101815	2109138	507.7586
Valeric acid	7.629	187381	3969495	506.1655

B

Name	RT	Area	Height	Conc.
Acetic acid	4.084	2140	32798	102.6179
Propionic acid	4.951	17	312	10.3264
Butyric acid	6.040	158	2765	12.2066
Valeric acid	7.620	424	8721	11.3809

C

Name	RT	Area	Height	Conc.
Acetic acid	4.084	2257	35030	108.0477
Propionic acid	4.951	17	283	10.3269
Butyric acid	6.040	23	503	11.5522
Valeric acid	7.629	73	1434	10.4520

D

Name	RT	Area	Height	Conc.
Acetic acid	4.084	4637	70124	218.0958
Propionic acid	4.879	15	202	10.2641
Butyric acid	6.027	46	387	11.6636
Valeric acid	7.611	32	676	10.3428

Figure S19. Quantification of SCFAs in Culture Media Samples Using the Headspace Method. (A) The standard solution contained four SCFAs—acetic acid, propionic acid, butyric acid, and valeric acid—each at a concentration of 500 μg/mL. (B) In the culture of *P. acidilactici*, acetic acid, butyric acid, and valeric acid were quantifiable, with a concentration of 2,376.3 μg/mL, 282.1 μg/mL, and 262.2 μg/mL (based on an input of 102.6179 μg/mL, 12. 2066 μg/mL, and 11.3809 μg/mL, respectively). Propionic acid was not detected in this culture. (C) Another *P. acidilactici* culture yielded acetic acid, butyric acid, and valeric acid at 2,694.4 μg/mL, 287.5 μg/mL, and 259.3 μg/mL (based on an input of 108.0477 μg/mL, 11.5522 μg/mL, and 10.4520 μg/mL, respectively), while propionic acid was below the limit of quantification. (D) In the culture of *L. rhamnosus*, the concentration of acetic acid, butyric acid, and valeric acid was 5,185.1 μg/mL, 276.7 μg/mL, and 244.7 μg/mL (based on an input of 218.0958 μg/mL, 11.6636 μg/mL, and 10.3428 μg/mL, respectively). Propionic acid was not detected in this culture medium.

Disclaimer/Publisher's Note: The statements, opinions and data contained in all publications are solely those of the individual author(s) and contributor(s) and not of MDPI and/or the editor(s). MDPI and/or the editor(s) disclaim responsibility for any injury to people or property resulting from any ideas, methods, instructions or products referred to in the content.
